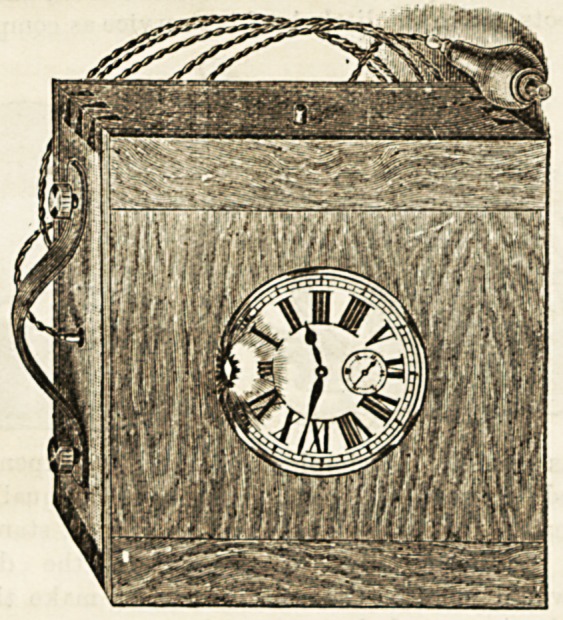# "The Hospital" Nursing Mirror

**Published:** 1899-10-07

**Authors:** 


### "The Hospital" Nursing Mirror.

Being the Nursing Section of "The Hospital."

[Contributions for this Section of "The Hospital" should be addressed to the Editor, The Hospital, 28 & 29, Southampton Street, Strand, London, W.C., and should have the word "Nursing" plainly written in left-hand top corner of the envelope.]

### Notes on News from the Nursing World.

### THE RED CROSS SOCIETY AND SOUTH AFRICA.

Lord Wantage draws attention at an appropriate moment to the operations of the Central British Red Cross Committee. The committee, which, it will be remembered, consists of representatives of the English Red Cross Society, the St. John's Ambulance Society, and the Army Nursing Service Reserve, are now in full working order, and are holding meetings in order to consider their plan of action in the event of war. Lord Rothschild, as a member of the committee, has already raised among his City friends the sum of £10,000, and this will form the nucleus of a fund for which, if war breaks out, an appeal will be issued to the public. The committee are already engaged in arrangements for chartering a vessel to be fitted up in the best manner possible, with every requirement and comfort, and with a complete staff of medical officers and of nurses supplied by the Army Nursing Service Reserve, to be used for the conveyance of invalided soldiers from the seat of war. It is also proposed to equip a railway train with the most approved ambulance fittings. But it may save some of our readers trouble and disappointment if we emphasise the fact that no applications can be entertained by the committee from nurses outside the Army Nursing Service Reserve.

### DO NURSES "LIKE" WAR?

There is an impression in some quarters that nurses "like" war. We are quite sure that they do not desire bloodshed, and the sacrifice of precious lives. But in the opinion of the matron of the Guards' Hospital "active service has the same attraction for a woman as for a man." And she confesses that in the event of war she should much like to go out. Her experience has been considerable, for she is the proud possessor of the Royal Red Cross, the Zulu War Medal, the Egyptian War Medal, the Ashantee Star, and the Khedive's Star. Miss Gray states that already some twenty nurses have sailed for the Cape: "They will not go to the front, but will be appointed to the base hospitals, which, as far as possible, will be stationed within near distance of the scene of hostilities. More nurses will have, of course, to go out; but at present, however, we have had no departmental orders on the subject. The nurses who have sailed have gone off very quietly, as it were, and it is only by comparing notes that we here at home can get to know who they are." As to the 70 nurses on the staff at the Guards' Hospital, the matron mentions that each nurse has a contingent of Army Hospital Corps men under her orders. And then, closing the interview as she commenced it, she said, "I've seen a good many years' service in South Africa, in Egypt, and on the West Coast of Africa, and I only hope it will come to my turn to go to South Africa again this [illegible] if there be war."

### [illegible]AINING FOR GREEK LADIES.

The Crown Princess of Greece will open the St. Sophia Hospital for Children at Athens in November. The hospital, which, when complete, will afford accommodation for 200 children, is beautifully situated about two miles out of the capital. The view from the balconies which surround each pavilion is one of the finest in the world. The whole of the buildings have been erected at the instigation and under the personal supervision of the Crown Princess. All the latest improvements in sanitation, electric lighting, ventilation, and asepticism have been carefully carried out. In order to render the structural details as perfect as possible, the most important London and German hospitals were visited at Her Royal Highness's commands. The primary object of the new undertaking is not only to afford to Athens a hospital for its sick children, but also a training school for Greek ladies wishing to become hospital nurses. In order to carry out this object, an English superintendent and English sisters have been engaged. The superintendent of the hospital is Miss Maud Calvert, who received her training at Guy's Hospital, and who, prior to her appointment at Athens, was lady superintendent of the General Hospital, Newark-on-Trent, and of the Seamen's Royal Albert Dock Hospital. Miss Calvert's long experience in the training of nurses and in general hospital management will doubtless prove of great help to her in organising her new work in Athens. The hospital is much in need of funds. Several pavilions must remain unbuilt until more people come forward to give their support to this most excellent undertaking.

### THE NURSING STAFF AT ST. THOMAS'S HOSPITAL.

It is well known that the number of applicants for training at St. Thomas's Hospital are far in excess of the number of probationers required. But the statement of the Treasurer, in his review of the year on Tuesday, will, perhaps, surprise some of our readers. For the 60 appointments it was necessary to fill during the 12 months, he said, between 1,800 and 2,000 ladies offered themselves. This is a striking proof of the popularity of the hospital.

### THE OPENING OF THE VICTORIA WARD OF YORK COUNTY HOSPITAL.

On Saturday, in the presence of a large representative assembly, Mrs. Thynne, wife of General Thynne, C.B., opened the new ward for children which has been added to the York County Hospital. There is accommodation for 18 cots, an isolation ward for three cots, a large day-room, ward kitchen, and bath-room. A feature which will be much appreciated by nurses, as well as by their little patients, is a large verandah. The floors of the wards and day-room are laid with polished Indian teak blocks, and the corridors, bath-houses, &c., with terazzo marble mosaic. All the walls are tiled to a height of five feet with pleasant French grey glazed tiles, and above they are finished with Parian cement. It will be recollected that some time ago Sir Henry Burdett paid a surprise visit to the old children's ward and reported very unfavourably of it, and also of the nurses' quarters. The Dean of York referred to this visit at the opening function, and mentioned it as one of the causes that decided the committee to build a new ward as a memorial of the Queen's Jubilee. The cost has been about £4,000.

### THE PRIVATE NURSES OF THE GLASGOW INFIRMARY.

EARLY in 1897 it was announced by the matron of the Royal Infirmary, Glasgow, that nurses for "private work" could be obtained on application to her. The terms quoted were 8s. per day, or £2 2s. per week, and attendance at operations £1 1s., the fees being payable to the nurse. The result of this experiment has, we understand, been entirely satisfactory, and there has been no difficulty in working. No efforts have been made to obtain publicity in any way, but the staff of ten nurses have had quite as much as they could do. Each member of the staff has the full benefit of her earnings, the only payment to the Infirmary being 1s. if the case lasts over a week. This small payment covers telegrams and incidental expenses during the whole time, however much it may exceed a week; and certainly shows consideration for the pockets of the nurses. As to residence, the nurses do not live in the infirmary, but lodge near at hand wherever they choose. During the period of nearly three years there has not been a single complaint lodged on either side.

### NURSES AND "BOARD WAGES."

IT is now more than three weeks since the Victoria Hospital for Children, Chelsea, was closed owing to an outbreak of diphtheria. The committee are doing everything in their power to overcome the difficulty, and it is hoped that the wards will be ready for reopening in a week or so. Meanwhile, the nursing staff are having an unlooked-for holiday. This sounds at first a great privilege, but enforced holidays are sometimes a heavy drag on a nurse's salary. The regular holiday takes a slice out of it, and to the railway and cab fares now needed again, must be added in many instances the money required for board, and sometimes for lodgings. So many nurses, unfortunately, have either no friends who can receive them for a second visit, or their friends are so situated that an extra one in the family for three or four weeks means an expense which severely taxes the resources of the housekeeping purse, and they feel it imperative to help out of their somewhat slender purses. Could not a "board allowance" be given to nurses during the holidays?

### WELSH-SPEAKING NURSES FOR WALES.

ALTHOUGH it may not always be practicable for the Welsh nursing associations to obtain nurses who speak the Welsh language, there is no doubt about the value of the qualification, other things being equal. The Trevor, Garth, and Vron Association, who have just lost the services of one nurse who can converse fluently in Welsh, have secured as her successor another who is also at home in the language. As the latter has enjoyed four and a half year's training there can be no question as to her professional capacity; and it is a distinct advantage that she will be able to talk in Welsh to those of her patients who have little or no knowledge of English. It is true that the Welsh people are much better acquainted with the English tongue than the English are with the Welsh, but the poor in the Principality especially appreciate being addressed in the language they love; and a nurse who can make herself understood in it has more chance of winning their confidence in her treatment.

### THE HASTINGS BOARD OF GUARDIANS AND THE WORKHOUSE INFIRMARY NURSING ASSOCIATION.

THE Hastings Guardians have been considering four questions submitted to them by the Workhouse Infirmary Nursing Association. The questions were as follows:—

"(1) Whether the board had met with difficulty in procuring an adequate supply of fully-trained nurses for the infirmary; (2) to what causes, in the board's opinion, the difficulty was attributable; (3) what would the board suggest to meet the difficulty; and (4) did the board consider it desirable that a central fund should be formed for training women under the Poor Law, and if such a scheme were found practicable, would the board subscribe under conditions which would be submitted later."

On the first point the guardians decided to reply that difficulty had been experienced in obtaining fully-trained nurses; upon the second they came to the conclusion that the reasons why nurses do not, as a rule, care for workhouse duty are that the cases are mostly of a chronic character, and that they do not like the style of living. As to the third, it was suggested that nurses should take apprentices; and respecting the fourth, the guardians answered to the effect that they are prepared to favourably consider the proposal to subscribe to a central fund for training women under the Poor Law, when the conditions are laid before them.

### OUR CHRISTMAS DISTRIBUTION.

THE attention of our readers has already been called to our distribution of clothing at Christmas. Of all gifts a useful warm garment that a patient can take home and wear is most useful and most valued. Flannel shirts and chemises are god-sends in cold weather; frocks and pinafores, babies' clothes, children's garments, anything and everything comfortable and pretty, for man, woman, or child, will be welcomed. Will every nurse who gives a kindly thought to the sick poor of London send us just what she can manage? We like each parcel to have the name and address of the sender inside, in order to acknowledge the gift in our columns. The parcels should be addressed The Editor, THE HOSPITAL, 28 & 29, Southampton Street, Strand, London, W.C., and should be marked "Clothing Distribution." They should reach us not later than Monday, December 18th.

### NURSES AT THE WEST LONDON HOSPITAL.

THE out-patient department at the West London Hospital has been closed for upwards of two months on account of extensive alterations and improvements. This nursing school offers special attractions to nurses from the fact that the hospital is not a medical school in the usual acceptation of the term. Post graduate students are afforded every facility for pursuing their studies, and they largely avail themselves of the excellent and varied bedside experience obtainable, but the dressings, &c., are completely under the care of the nurses. The nurses also have useful courses of lectures, and the result is that the number of candidates wishing to become probationers is far larger than can possibly be accepted. Promotion too is made from the staff whenever possible. Sister Devonshire, for many years one of the most valued and capable of the staff, has unfortunately been compelled to resign recently on account of ill-health. She is an old "London" nurse, has earned a high reputation for skill and efficiency, and her departure is regretted by everyone. Her post has been taken by the night superintendent, Sister Reay, who has been in the service of the hospital for a long time. She will enter on her new duties upon her return from her holidays, when Nurse Stollard, also on the staff, will take her place as night superintendent.

### MEALS IN THE SURGERY.

The Haslingden Board of Guardians do not get on very well with their nurses. There have been numerous resignations; and, though there may have been faults on both sides, it is clear, from the discussion at Pikelaw, which is fully reported in the Accrington papers, that the accommodation provided for the nurses is of a very unsatisfactory character. For example, Miss Perkins, one of the nurses whose resignation has been accepted by the Guardians, complained that she had to take her meals, and to spend her recreation hours, in the surgery. It is stated that a new room is being got ready, but the protest, "that surely the long hours of duty among the sick gave a nurse sufficient smell of drugs and medicines without her eating her food in the surgery," is exceedingly reasonable. Such an arrangement ought never to have been thought of.

### NEW NURSING HOME AT WOOLWICH.

Last week the Jubilee Commemoration Nurses' Home, which has been erected under the auspices of the Woolwich, Plumstead, and Charlton Nursing Association, was opened by Mrs. Escreet, wife of the rector of Woolwich. It is situated in nearly the centre of the district, and, thanks to the labours of Mr. and Mrs. Grinling, the cost of the undertaking—£2,500—has all been met, save a balance of £250 for the furniture.

### THE SALARY OF A NURSE-MATRON.

At a recent meeting of the Leominster Cottage Hospital Committee, the Chairman stated that they had advertised for a nurse-matron, and had received 50 applications. Their choice, he said, had fallen upon Nurse Ellen Harrington, of University College Hospital, and he congratulated the committee and the trustees upon her qualifications and experience. Miss Harrington has been at University College for 30 years, and holds the Queen's Badge of Merit. Yet she is only to receive a salary of £20 a year. As this was the salary advertised, the applicants for the post knew what to expect; but it is a curious and significant fact that, while a nurse-attendant, who is not as a rule trained, can easily obtain £30 a year, the services of a nurse-matron, fully trained and with valuable experience, may be secured for 50 per cent. less.

### BRUTAL ATTACK ON A NURSE IN EAST LONDON.

A disgraceful attack was made on Sunday night on one of the nurses of the East London Hospital, Whitechapel. According to the *Daily News*, she left the hospital about ten to walk to the home in Philpot Street. "On the way she was stopped by two men, one of whom asked, 'Have you got any money?' The nurse was naturally much frightened, and tried to return to the hospital, but she was seized by her cloak, and in the struggle she fell, or was knocked down. Her screams attracting attention, her assailants—who have not been arrested—made off, and the nurse exhausted, but not much hurt, at length got back to the hospital." It appears, however, that this is by no means the first occasion on which the nurses attached to the institution have been molested in Oxford Street, and in future an extra constable is to be posted near their entrance to the hospital. The least that the authorities can do is to protect the nurses from insult and robbery by violence.

### NURSES AND THE AROMA OF THE TANYARD.

It is stated in the *South London Press* that some of the nurses employed at the Bermondsey Workhouse have suffered from indisposition due to inhaling the odours from the adjacent tanneries. Dr. Dixon, the medical officer of health, does not consider that the nurses have "any serious cause of complaint," but several members of the vestry affirm that the smell is highly objectionable, and the matter has been referred to the Sanitary Committee for investigation. We agree, however, with our contemporary, that in any case pungent smells can hardly be dissociated from tanyards, and that "if it became a question of the retention of the tanning industry on the one hand or the workhouse nurses on the other, the nurses would have to go to the wall." Bermondsey, it is truly contended, could not do without its tanneries; but we do not think it will become necessary to adopt the suggestion "that the authorities of the workhouse should rear nurses in the locality, who, like one of the members of the vestry, would wax fat and ruddy upon the aroma of the tanyard."

### "NAE GOD IN DUNFERMLINE."

A correspondent vouches for the accuracy of the following incident, which was told her by the nurse to whom it happened. She was night staff nurse in a large Scotch hospital. In her ward was a small boy whose leg had been amputated. When the ward was crowded, and he was convalescent, he was often put in another small boy's bed to make room. "He was an obstreperous child," continues our correspondent, "and used to try and kick his companion with the 'stump.' Then there were terrible rows. The night nurse was often angry with him, as were the day nurses, and once or twice the visiting accident policeman told him to be good, or he would take him away. One night when the nurse came on duty she found him crying, and on asking him what was wrong, got this reply: 'When I am hame wi' my mither, she says God sees me, and when I am here the "perlice" sees me, and I wish I was wi' my grannie in Dunfermline, for there's nae God or nae "perliceman" in Dunfermline.'"

### SHORT ITEMS.

The proceeds of the fancy fair on behalf of the Crieff Nursing Association reached the splendid total of £1,384, and that amount has been received by the trustees.—The Toynbee Nursing Guild has again resumed the winter's work at Toynbee Hall. Dr. W. J. M. Ettles is giving a series of fortnightly lectures, and on alternate Wednesdays Nurse Jessie Mackay (who has just been appointed lecturer on health and hygiene to the London School Board) lectures on tuberculosis.

### Clinical Lecture on Two Cases of Placenta Prævia.

Delivered to the Nurses in the Glasgow Maternity Hospital by Robert Jardine, M.D., &c., Physician to the Hospital.

As we happen to have two cases of placenta prævia in the hospital to-day we shall discuss them. While these cases are alike in some respects, in others they differ widely, and they should therefore help to fix the subject in your minds.

Case I.—Single; i. para, æt. 19; about 6½ months pregnant. She came to the hospital a few mornings ago with the history of a sudden onset of hæmorrhage while in bed. The loss of blood had not been very great. On examination the os was found slightly dilated and the edge of the placenta could easily be felt extending partially over it. The treatment adopted was to firmly plug the vagina. When I saw her some hours later uterine contractions were coming on. There was no bleeding, although it was quite evident the cervix was being dilated. We decided to take the plug out about three p.m., but shortly before that the uterine contractions became so strong that expulsion of the plug began. Dr. Wilson withdrew it, and labour terminated by natural efforts in a few minutes. There was no bleeding before or after the birth of the child.

Case II.—Married; xiv. para, æt. 39, seven months pregnant. Dr. Craig, the lady resident at the West-end branch of the hospital, was called to this patient early on Sunday morning. She found that there had been a considerable loss of blood, and that the patient was very collapsed. There had been several slighter hæmorrhages during the last three weeks. This attack had come on while she was in bed.

The patient had suffered from chronic bronchitis for several years, and her constitution was evidently undermined. Dr. Craig plugged the vagina and brought her to hospital. On admission at half-past seven a.m. she was extremely blanched, and her pulse very feeble. The vaginal plug was removed, and the largest-sized Barnes' bag inserted. She was freely stimulated with hypodermic injections of strychnine and spirits by the mouth. When the bag was inserted her pulse became imperceptible for a short time.

When I saw her shortly afterwards the bag had been expelled during a fit of coughing, but there was not much bleeding. She looked to me as if she would in all probability collapse and die in our hands during delivery. Before doing anything we injected under the skin in the right mammary region fully a pint and a half of saline solution (one teaspoonful of common salt to the pint at a temperature of 100). On examining her vaginally I found the os would admit three fingers easily, and that the placenta was laterally situated. Without giving her chloroform I quickly turned the child by the bipolar method, and brought a foot down. The body and aftercoming head were brought through slowly so as not to tear the cervix. The placenta quickly followed the child, and fortunately there was no post-partum hæmorrhage. The foot of the bed was raised, and the patient was surrounded with hot bottles to keep up her body temperature. She was freely stimulated and given plenty of fluid diet. Contrary to our expectations, she has made a wonderful recovery, and is anxious to go home on the tenth day.

These two cases are very instructive. They represent the two extremes of the child-bearing period. The first one you notice is a primipara. This is rather unusual, especially in so young a primipara; and then, again, this was her first attack of bleeding. As a rule, you have a history of several attacks before the labour comes on. In this case the edge of the placenta actually encroached upon the os, so that as regards position it was in a worse situation than in Case II., and yet the bleeding was never great. Case II. was a typically bad one. The woman's constitution is enfeebled by chronic bronchitis and from bearing so many children. Her heart is undoubtedly dilated. The history is the usual one, of several slight attacks of bleeding about the seventh month of pregnancy, then a very severe one. No attention is paid to the slight attacks, and assistance is not sought until she has almost bled to death.

This form of ante-partum hæmorrhage is called unavoidable. The placenta, as you know, is wholly or partly attached to the lower uterine segment, *i.e.*, to the part of the uterus which stretches during dilatation. The placenta cannot expand to keep pace with the expansion of its site of attachment, so separation is bound to occur. As separation occurs the utero-placental sinuses or blood-spaces are opened and the blood pours out. In the other form of ante-partum hæmorrhage, known as accidental, the bleeding is caused by separation of the placenta but its site of attachment is normal, *i.e.*, somewhere in the uterus above the lower uterine segment. In both cases the bleeding is from the torn uterine sinuses, but in placenta prævia they are in the lower uterine segment, while in accidental hæmorrhage they are above this segment, *i.e.*, above the situation of Bandl's ring. There is a small amount of blood lost from the torn placenta, but it is of little consequence.

Placenta prævia is a dangerous condition, but if the patient is seen in time and is properly treated 95 per cent. should be saved. As regards the chances of the child they are small. If the placenta is much separated it will die from want of purification of its blood. It is usually premature and it generally has to run the risk of artificial delivery, so that the majority do not survive. About one-third of them are born alive.

Treatment: A nurse's duty is to send for medical assistance at once on diagnosing placenta prævia, no matter how small the amount of bleeding may be. Until assistance arrives she should keep her patient lying perfectly still. It may occasionally happen that a nurse is so placed that medical assistance cannot be obtained. She must then do the best she can for the patient. Under such circumstances there are various things she may do. In a case where the os is only slightly dilated, and the bleeding is going on, the best treatment is to thoroughly plug the vagina. It will stop the bleeding and also assist dilatation of the os. You see how admirably it acted in Case I. The plug must of course be aseptic. If the os should be fairly well dilated the membranes should be ruptured and the uterus stimulated to contract by rubbing it so as to force the presenting part down. If the breech presents a foot should be drawn down. Delivery should not be hurried by traction on the legs. In a transverse case the hand should be passed in and a foot brought down. If it should be a case of central or complete placenta prævia the fingers should be passed round the edge of the placenta and the membranes ruptured, or, if this cannot be done, the fingers should be forced right through the placenta and the leg be brought down through the opening. You will understand that I am only advising you how to act if you are forced to do so by being unable to get medical assistance.

After delivery there is considerable risk of post-partum hæmorrhage. The torn sinuses lie practically open in the top of the vagina, and you can easily understand how they may bleed. The hot douche is not so useful in these cases as in ordinary post-partum hæmorrhage. One of the best ways to control the bleeding is by compressing the cervix and uterus between one hand in the vagina and the other over the fundus.

If the patient has lost a great deal of blood, saline injections are of great use. In Case II. we gave a large one beneath the skin before delivery, and I firmly believe if this had not been done the patient would have died. A nurse cannot do this, but she can give one into the rectum. This is not so good a method, but it is very useful. The patient's head must be kept low, and her body temperature must be kept up by placing hot bottles about her. She will also require plenty of fluid food and stimulant. It is surprising how quickly these patients improve. In Case II. you must all have noticed this. They run a much greater risk of sepsis than ordinary cases. These two cases, however, have escaped that risk.

### Proposed Cottage Hospital at Moose Fort, Hudson Bay.

### RED INDIAN PATIENTS.

Even in these days of universal philanthropy it may seem strange to read of a proposal to found a cottage hospital for Red Indians, and some may be disposed to question the necessity for such a scheme. But the so-called hospital is to be only a primitive log house, such as usually accommodates the family of a Hudson Bay Company employé, and it dare not aspire at present to the possession of a resident doctor or a nursing staff. Perhaps a home for the sick and aged would be a better definition for the proposed institution. In order rightly to understand the desirability of such a home it is needful to know something about the present condition of the people living round Hudson Bay. Let me picture a few scenes from life, showing sickness as it now is amongst the Cree Indians at Moose Fort, situated at the southern extremity of the bay.

The first I take is in the late fall. The frost has already destroyed every vestige of green grass and hardened the earth like iron. The early snow has melted away, only to reappear later, deeper and more lasting. The river flows dark and sluggish, as if feeling already the grasp that will soon bind it fast in icy chains for six long months. The Indians have heeded the warning and departed to their isolated hunting-grounds in the shelter of the fir woods which clothe the diocese of Moosonea for hundreds of miles; the only traces of their summer habitations on the river bank being the groups of bare tent-poles still standing amidst the circles of stones which had served to weight down the coverings.

One tent remains, but that has been moved back in order that it may be protected by the bush from the bitter winds that sweep across the open with a deathly chill.

Henry Lisk, the old hunter who lives here, cannot leave yet, for his son, Sammy, is dying of consumption. For months the poor lad has been getting gradually weaker, and his struggle for breath is pitiful to see. But such is his hold on life that it seems probable he may linger some time yet. His parents care for him in their ignorant way. He receives a small daily allowance of milk from the Company, and his mother offers him from time to time a very doubtful-looking liquid in which a rabbit or bird has been boiled; he turns from it as he did from the fried goose and duck on which the rest of the family had been feeding through the weeks of the autumn flight of those birds.

The missionaries bring little things to tempt his appetite, but he is only one amongst several needing help, and they have as yet no conveniences for affording constant, systematic care.

His surroundings are of the very worst for his case. The keen wind penetrates the cotton tent, the smoke from the burning logs in the centre aggravates his cough. For weeks he has been unable to lie down, and can only find comparative ease by kneeling up, leaning upon a wooden box for support. He wears a flannel shirt given by the mission, and is wrapped besides in an old blanket coat and a rabbit-skin rug. The parents and younger children live, night and day, with him in this small space, so that on the whole it is well that the outer air has such free access, in spite of the cold!

Is it any marvel that Sammy's death one bitter morning brings a sense of relief to all, even to his sorrowing parents? The very next day the emaciated form is laid to rest, the few possessions of the family are packed into the birch-bark canoe, and the Lisks hurry away, many miles up the river, where they can catch fish, flesh, and fowl to make up to them for the short commons on which they have all subsisted during the time that poor Sammy's illness obliged them to remain in the settlement.

Yet another case of lung trouble, the commonest ailment amongst these poor folk, exposed to all the changes of that inclement region. Gideon has been given the use of a log hut, where he may live with his old wife; but what a place for a sufferer! The only furniture is a wooden box and a tiny stove that has been lent them. The sick man lies on the board floor rolled in a rabbit-skin rug, with not even a pillow on which to rest his aching head. As winter closes in he will have to share this refuge with any to whom the Company may see fit to grant a similar privilege.

Old blind Harriet may crouch in one corner, with no one caring enough for her to wash, from one year's end to another, the blanket which she wears as a shawl. A sick child may be laid in another corner, his fretful wail disturbing the rest of the others, whilst his mother and little brothers carry on the business of daily life with hardly room to turn round. There is, naturally, no certainty that food sent to this hut reaches the right person, and certainly no scrap could be saved for him, even for an hour.

Again, here is old Granny Pott, dying of old age; yet she lies on the ground in her wigwam shivering with cold. Her granddaughter, Jane, is supposed to be in charge, but she is a giddy girl, and will wander about for hours with her friends, and the old woman is so fearful in her lonely helplessness of taking fire from the embers on the ground that she insists on the fire being beaten out before the girl leaves, preferring cold to the risk of being burned to death. Warm garments had been given her by friends, but her relatives, thinking her life nearly ended, and each fearing that someone else may secure the best of the prey, have heartlessly taken them from her. Soon she sinks into a state of unconsciousness; death comes to her, too, as a merciful release.

How sorely needed is this home into which it is hoped the aged and dying will be gathered, their wants attended to, and their sufferings alleviated by gentle hands. The arrangements will be of the simplest, country food, *i.e.* fish, rabbits, &c., used as much as possible, but the food will, at any rate, be wholesomely cooked and the patients kept clean.

The existence of such a place will obviate the necessity for the families of the sick remaining on the island, half-starving, and barely concealing the impatience with which they await the death which will set them free to go off to the hunt which means their livelihood.

The only medical practitioner on the Bay is the doctor appointed by the Company, for whom board and lodging is provided with the other gentlemen at Moose Fort. But there is a grand opening for a young student who is willing to face the monotony of northern life that he may mitigate the sufferings of his countrymen and the Indians, and the time may shortly come when things will be sufficiently advanced for a self-sacrificing nurse also to endure the hardships of life in this lonely district, and do her part among the suffering natives.

### The Nurses of Guy's Hospital.

A Chat with the Matron. By Our Commissioner.

### RECENT CHANGES.—PROGRESS OF THE PENSION FUND.

It was a few days after the Matron returned from her holiday that I saw Miss Nott Bower at Guy's, and asked her to tell me about the changes which have taken place during the hospital year.

"So far as the nurses are concerned," she replied, "the most important event that has happened since last October has been the alterations in the hours of duty. We believe that Guy's has now the shortest hours of any of the London hospitals."

"What is the average number per day?"

"Eight hours and three-quarters. Our average was ten hours and a half, so that the reduction is a very substantial one."

"And how do you manage it?"

"Not by diminishing the time of duty per day, but by giving whole days and half days off. We give two whole days and three half days once a month. This enables a nurse to start at seven or eight on Saturday evening and come back at ten on Monday evening, and gives her an absolute rest. As to the half days, they are available for dramatic and musical performances or short visits to friends."

"How many nurses have you had to add to your staff in order to make the reduction?"

"Thirty extra probationers. These were added entirely because of the reduction of hours, and not at all because of an increase in the work. The number shows that a complete rest is given."

"Are you satisfied with the result?"

"Extremely well satisfied. It has proved of great advantage to the health of the nurses here, and some other hospitals have since diminished the hours of duty."

"Do the nurses know beforehand when they will get their days and half days?"

"Yes, the system works automatically now. There is a time-table in every ward, and each nurse can tell not only the day, but the minute of the day, when she will be off duty."

"What other innovations have you introduced in the year?"

"We have started training in maternity work, and in massage."

"How many examinations have been held?"

"Only one in each subject so far. At present the accommodation for training in midwifery is limited to six; but it is intended to increase the number."

"A change in a different direction," continued the Matron, "has been the formation of a Nurses' Recreation Society. Our recreation ground and club house at Honor Oak Park will be ready for us early in spring, and by that time the society will be in full swing."

"What is the extent of the recreation ground?"

"About an acre and a quarter. It is very charming, and will contain three tennis courts and two croquet lawns. We are just starting a new library, which will be managed by the society; and the choral society, cycling club, and tennis club will be amalgamated with it as soon as the club is opened. Our concerts have been very successful, and we have a professional conductor who is much liked."

"Do you make money by them?"

"We took £30 at two last year, and bought a piano with the profits."

"How many years is it since the governors of Guy's decided to federate with the Royal National Pension Fund?"

"Eleven. Previously to that time there was a small pension assured to the sisters only at the age of sixty. Up to 1893 the age was fifty-five, but when, in that year, we started a pension fund for the Trained Nurses' Institution, we made it fifty. The Royal National Pension Fund did not at that time recommend the reduction of the age to fifty, but they afterwards recognised the advantages. I feel strongly that nurses should be able to retire when they are fifty."

"I think it is compulsory for your nurses to join the Pension Fund?"

"Yes, three years ago we made it compulsory for Staff nurses to join. Probationers are allowed to do so, and at the end of their training, if they remain with us, they must. The idea is, of course, that every nurse should provide for the future. The minimum amount of her policy is £7 10s., and the hospital takes out for her with the Royal National Pension Fund a policy on the returnable scale (Table B) for a pension of £11 5s. If they can afford it, nurses very often avail themselves of the chance of joining for a larger sum."

"What do you reckon that the amount of the minimum pension will be?"

"With the bonuses, I believe the authorities of the Fund reckon that, roughly speaking, at fifty a nurse will be entitled to £25 a year. But we cannot tell exactly until some of the policies fall due."

"When will that be?"

"In about five years."

"Has any objection been offered to the compulsory application for a policy?"

"At first some of the nurses did not like it, but objections are never offered now. Why should there be, seeing that all the money is returnable? If a nurse retires from work, or marries, or from any cause desires to withdraw from the fund, she is entitled after two clear years have elapsed from the time of entry to receive the amount of the premium she has paid back again, with Saving Bank interest."

"According to the Pension Fund rules?"

"Exactly. Moreover, suppose that a nurse leaves the hospital after five years' service, the Governors give her the policy they have taken out for £11 5s. They do not undertake to do so, but if she has complied with the rules laid down she has the benefit of the policy effected by the hospital on her behalf."

"What becomes of money left in the Fund?"

"It is devoted to the maintenance of invalided nurses. Now and again a sum falls into the Fund, and very useful it is to the matron, certainly."

"You attach great importance to the Pension Fund?"

"The greatest. I have known of so many cases of old nurses coming to the workhouse. No deserving nurse who has done good consecutive work at Guy's should fall into such a position."

"Do many probationers leave at the end of their three years' training?"

"Of those who get their full certificate not more than two or three move on. As a rule, they are glad to remain on the staff either of the hospital or the Institution. It is very seldom that a nurse leaves us, except to get married or to fill an appointment."

"You speak from a long experience of Guy's?"

"Yes, I have been here eighteen years, with the exception of one year, when I was matron at Huddersfield."

"Do the nurses prefer the hospital or the Private Nursing Staff of the Institution?"

"Some prefer the bustle and excitement of the hospital and others the quietness of family life, and some train for the hospital, and some for the institution. They get more money at the institution. The salaries rise to £40, as compared with £30 in the hospital. Then the profits of each year are divided as bonuses, which vary with length of service from £8 to £40. With extra fees, as in massage or mental cases, or cases involving extra fatigue, the amount runs up in the year to quite a high figure."

"Is there a constant demand for the services of the private nurses?"

"We have half a dozen applications more than we can accept almost every day. There are nearly a hundred nurses in the institution, but we cannot increase the number rapidly with the present system of training. No nurse can leave the hospital for the institution until the expiration of her three years."

"You are building a new nursing home?"

"Yes, and it is very badly wanted. Our dormitories are comfortable and well ventilated, but they are not what they should be."

"How many bedrooms will the home contain?"

"Two hundred. There will be a dining-hall to seat 140, a fine sitting-room, and rooms in which the nurses can meet their brothers or fathers. There may also be a swimming bath. As you are aware, Mr. Raphael gave £20,000 towards the erection of the nursing home, but it will cost much more than that sum."

"You have just renovated and re-opened some wards?"

"Yes, the 'Cornelius,' 'Lydia,' and 'Dorcas' have been thoroughly renovated and the 'Queen Victoria' ward has been re-opened. There is increased accommodation to the extent of forty beds, which means an increase of ten to the nursing staff. The average in the hospital, however, is two and a half patients to each nurse, not four."

And then Miss Nott Bower kindly conducted me over the Cornelius ward, in which I observed many novelties.

### Across the Seas.

### NURSING IN THE BAHAMA ISLANDS.

By the Matron of the Government Hospital.

The Government Hospital for all the Bahama Islands is situated in the City of Nassau. It was built in honour of the Queen's Jubilee, and is called the Victoria Jubilee Hospital. It contains forty beds, twenty male and twenty female. It is a perfect and smart little hospital, with a delightful verandah running round it, and a nice little garden in front, where the roses and other flowers grow in winter, as well as in summer. It stands in its own grounds, and the view of the sea is very fascinating. The waters are as clear as crystal, and show ever-varying gradations of colour; no language can describe their tints, and I do not think that any pigments could really reproduce them. The moonlight here is also very beautiful. Large parties go cycling and boating by moonlight. The surgery is nicely fitted with cupboards for the instruments, a good operating table, and satisfactory arrangements for sterilising the dressings, which is invariably done as carefully as in any large London hospital. The walls are whitewashed and the floors are scrubbed.

The greater part of the work is surgical, and some very good operations are performed, and they are very successful. Terribly contused wounds and scalp injuries recover marvellously, generally healing by first intention. Most of the patients are alcoholic, and take ether very badly. Accidents resulting from alcoholic assault and battery are very common, often assuming the form of severe gunshot wounds and other injuries. They are usually brought in by the police, and end in a few weeks in hospital for the victim, and a few months in gaol for the offender.

There is an infirmary containing four wards for convalescent patients. Here disabled and old men and women who cannot work and have no place of refuge can remain for years in peace and comfort. There is also a male and female lunatic asylum. The inmates are nursed on the principle of straps instead of a padded room; they do not hurt themselves, so I conclude that there is not much to choose between the two system. The average number of in-patients is 150.

An English resident surgeon-superintendent performs all operations and manages everything with the help of a qualified native assistant and a storekeeper. As I am the only trained nurse, I have entire charge of the nursing department. I am assisted by two native ladies; one assists me in superintending the hospital, and the other acts as housekeeper. We each have our own rooms and our own servant.

The ward nurses are all black natives with the exception of one, "Mannie," a Chinaman; Mannie has been in this hospital 23 years. He is a really good nurse and very trustworthy; he can read and write, and speak English fairly well.

The black natives are very lazy and dirty, very superstitious, and yet easily convinced that what is done is for the best. They have great faith in the doctor, and they give very little trouble except on the subject of soap and water and general untidiness. They will quote instances of how people have died through taking a bath, and some of them would rather leave the hospital than be washed. They live almost entirely on fruit, hominy, and fish, and as it is seldom cold they require very little clothing. They are generally respectful to their superiors, happy-go-lucky in temperament, and have very little conscience.

The sick patients on special diet are fed on tinned milk, arrowroot, bovril, and soup, and stimulants as ordered by the doctor. When they are put on ordinary diet they have hominy, meat, turtle, fish, rice, meal, bread, &c.

Fresh milk is scarce and expensive, and therefore it is ordered only in very special cases.

Nassau is a fashionable health resort for Americans, and in the winter months every hotel and boarding-house is crowded with visitors, and yet, strange to say, there is not one trained nurse available for private nursing. In sickness the Europeans and Americans are absolutely dependent upon each other for nursing.

In the summer Nassau is very dull from a society point of view, but the roads are very good for cycling and the water is nice for bathing. So, in spite of all difficulties, with continuous sunshine, blue skies, and a breeze from the sea, one can be very happy nursing in the Bahamas.

Most of my friends in the nursing world will see by this that I have survived the hurricane that has passed over the islands with great loss to life and damage to property.

### Presentations.

Tynemouth Jubilee Infirmary.—Last week the nurses and patients of the Tynemouth Jubilee Infirmary presented the late matron, Miss Hill, with an exquisite silver teapot and stand. The presentation was made by the senior nurse, who described it as a voluntary token of affection and esteem. Miss Hill, in the course of a suitable response, expressed her warm appreciation of the gift.

### Pictures from Italy.

### NURSING AT THE HOSPITAL AT SIENA.

By a Travelling Nurse.

The brethren of the local *confraternità* had set down the ambulance before the door of the hospital, and we had to wait till the inmate had been lifted out and the small crowd of onlookers dispersed. We watched the weird-looking brethren as they carried the ambulance away across the piazza, each member wearing a robe of linen and a cowl, which was drawn right over the head and face. Their dark eyes looked hollow and mysterious as they peered through the two holes left for the purposes of sight. It is the mission of the *confraternità* to perform all the duties connected with the carrying of the sick and the burial of the dead.

When they had disappeared in solemn procession we turned again to the hospital, and found no difficulty in gaining admittance. The porter, with that happy irregularity native to Italy, left his post at the door and elected himself guide. He was extremely interested to learn that we were English *infirmiere*, and said that if we would wait till the nurses had finished dinner, they would be glad to talk to us. Meanwhile, there were the frescoes, the catacombs, and the chapel of that ever-blessed woman, St. Catherine of Siena.

It is said that the hospital was founded in the ninth century by the Beato Sorore, but nobody knows much about the founder or founders beyond the name. It is built on the edge of a steep hill, and down below the wards the rock is honeycombed with curious rooms or cells, partly built, partly hollowed out of the hillside. The architecture is simple and massive, but the wards are finely proportioned with beautiful ceilings, heavily beamed and painted. The hospital was once a veritable museum of Sienese art, but now its finest treasures have been placed in the picture gallery. In the fine entrance hall, called the Pellegrinaio, or Pilgrims' Ward, remain eight large frescoes of the fifteenth century showing incidents connected with the existence of the hospital. One fresco depicts a patient who is bleeding from a severed artery, and I could not help thinking that the medico and attendants standing about would never get that ligature on in time. But though open to criticism as to treatment, the frescoes form a beautiful mural decoration, and one rejoiced that the lingering mediæval spirit lent itself to making beautiful the dwellings of the sick and infirm.

That strongest and perhaps greatest of women saints, Catherine of Siena, is intimately connected with this building. During the terrible plague, which almost depopulated Siena in the fourteenth century, she worked day and night in the wards, where the stricken lay heaped on mattresses and blankets so that "foot could not be set between." Nurse, friend, and intercessor, she soothed the dying and closed the eyes of the dead. In the night, when exhausted by fatigue, she would sometimes steal down the many steps into the vaults and pray, and perhaps rest a little in a tiny dark chapel now called Santa Catarina della Notte. There is a quaint picture of her in the town showing the saint bringing back to life a man who has died of the plague.

The nursing of the sick is superintended by the Sisters of St. Francesco di Paolo. They wear a costume like the Sisters of Mercy, with blue stuff gowns and large, flapping head-dresses. One dear old thing could hardly drag herself about, and we thought her age should have prevented active duty. She was, however, most anxious to talk about English hospitals, and inquired especially about a university which she had heard was in charge of a sisterhood. The wards are large, light, and airy, the linen, as usual, beautifully clean and hand-woven; but there was a lack of nicety and surgical cleanliness throughout the place. The operating-room, though not wanting either in fittings or instruments, looked the reverse of antiseptic, while the open doors of the cupboards which contained lint, bandages, &c., revealed a state of disorder almost indescribable. After this it is only fair to add that the hospital is said to have a good record for surgical work, and that the paying wards lately instituted for surgical cases are a complete success.

The patients in the ordinary wards looked extremely picturesque. All the women wore quaint little linen caps and coloured bed-jackets. The men had a kind of loose smock and cotton night-caps. Later on we saw the dinner served. The helpings were abundant, and consisted of a kind of rissole made of rice and minced-meat, with the usual accompaniment of red wine. The rissoles looked appetising and might have been nourishing if made with fresh meat; we suspected, however, that it had previously served an apprenticeship to the soup.

One incident occurred that somewhat astonished us. We were listening to our guide's description of a fresco in St. Peter's Ward when the *infirmiera*—a capable-looking woman —requsted us to move on, as some of her cases were very ill, and talking disturbed them. It was the first time in our Italian experience that noise of any kind had been considered as deterrent to the recovery of the sick. Usually it is supposed to keep their spirits up.

Presently the porter got hold of some disengaged *infirmiere*, and put us all on a balcony at the end of the Pelligrinaio. There was a lovely view stretching miles away over the Tuscan valley, and the vineyards were flecked with dusky patches showing where the purple grapes were ripening among the leaves. The *infirmiere* belonged to the ordinary peasant class, but appeared more intelligent and more business-like than the usual Italian attendant. They wore jackets of white linen made after the pattern of the women patients' bedgowns, and large aprons of the same material. This preponderance of linen in their dress gave them a clean and wholesome look, which we had found notably lacking in other places.

They were eager to hear about English hospitals, but naturally enough could not conceive of a nursing system in which the nuns have no place, and their interest died out after a few questions. On their own part they were more than ready to give us information. They were proud of their hospital, which they regarded as extremely progressive, mentioning the wards for paying patients as an example. The routine of nursing has the same features of irregularity as other Italian institutions. The *infirmiere* lodge outside the hospital and find their own food, receiving a daily wage of one lira and a-half. They evidently think that they are well paid, and, considering that the average wage of trained workwomen in the towns of Italy — dressmakers, fine starchers, &c.—seems about one lira per day, the authorities at Siena must be accounted generous in the matter of payment.

It must be borne in mind that living in Siena is extremely cheap for everybody except the English or American visitors, for whom the Sienese, like other Italians, have special prices. It is easy for the native to live well at a very small charge, especially as the climate is productive of good digestions. Chicken, eggs, vegetables, fruit, and various forms of maccaroni cost one-third less than in England, and there is always the good red wine—quite different from the thin French clarets—grown on volcanic soil, and possessing a body and tonic properties almost equal to Burgundy. On the other hand, meat is bad, and inspires the belief that the economical Sienese, having worked his oxen in the fields till extreme old age, prevents natural death by killing them for food. Mutton s unknown—no self-respecting Italian will eat it—and the veal is almost as tough as the beef.

When our round finally ended at the door of the hospital, we found a long row of people seated on the stone bench outside, patiently waiting for our friend the porter. "It is nothing," he said, when we expressed our sorrow at having detained him so long from his duties. Half an hour, more or less—what did it matter in Siena?

### Side Lights on Asylum Nursing.

### A VISIT TO WOODILIE.

I wonder how many of our hospital nurses have any idea what nursing in a modern, up-to-date asylum is like?

No doubt many of those who read The Hospital from week to week will have taken note of the interesting series of papers and letters on the medico-physicological question as applied to asylum workers, which appeared some little time ago, and will have read the correspondence on the subject. But I fancy very few realise what an important branch of nursing mental nursing really is, or under what pleasant conditions it is carried on in the asylums of to-day. These conditions are exemplified in the best and most modern way at the beautiful asylum of Woodilie, provided by the liberal City of Glasgow for its mentally-afflicted poor.

Situated ten miles from the bustle and noise of the busy streets, it stands on a hill just above the railway junction of Lenzie, and commands a grand view of hills and woods, a lovely prospect which must surely have a soothing influence on many a weary brain.

The buildings, or rather series of buildings, are in the midst of grounds comprising over 500 acres of gardens, woods, lawns, and farm land, through which run real country lanes, with banks and hedges. It is a colony in itself, for it raises on its farms a good proportion of all the food required for the large number of inmates and the attendant officers and employés. It has its own blacksmiths, carpenters, and wheelwright's shops, its own "abattoirs" and mills, and even its own railway for bringing in stores of coal, &c., from the junction.

A well-kept avenue leads up to the entrance, before which stretch out wide lawns, brilliant with beds of autumn flowers, and beyond appear tennis-courts, bowling-greens, croquet lawns, and cricket grounds, bordered by shady vistas of winding walks.

On entering, it is impossible not to be struck with the peaceful feeling of the place, and with the quiet order which prevails everywhere. Our preconceived ideas of lunatic asylums receive a rude shock. Here are no solitary cells, no padded-rooms, no gloomy bars and iron bolts, no resounding calls, and shrieks from wild-looking, frantic lunatics, and no closely-locked doors. Instead, all is sunshine and brightness, open doors, and the quiet hum of busy and happy life. Men and women, solitary or in groups, are seen walking in the long corridors allotted to their several divisions, or sitting in the bright dayrooms, working or reading. The greater number are busily employed in the various branches of work needed to carry on the daily life of so large a family.

The next thing to be noticed is the cheerful and contented expression on the faces of the majority of the patients. Here and there a sullen, a distinctly morose expression is seen, but it seems to be the exception.

Another surprise awaits one on entering the vast dining-room. Stories of how lunatics are only allowed spoons or fingers wherewith to eat their food, from tables devoid of cloths, pass through one's mind. Here are tables daintily covered with clean white cloths, and supplied with the full complement of spoons, forks, and knives.

Under the enlightened and humane system of treatment carried out at Woodilie most fully by the medical superintendent and his able assistant, the old method of restriction is entirely abolished; no force or restraint is allowed in any form. The nurses are trained to manage and control their charges without holding them in any way—even when some poor creature is seized by a sudden delusion which prompts him to start up and flee from some unknown horror.

Instead of the old system of control, and fear, and restraint, too often accompanied by punishment, they are now treated more like sick children who are wayward and peevish, whom contradiction will only make worse, and if controlled by kindness would learn to control themselves. The system is one of ever watchful observation on the part of the nurses, who must, as far as possible, try and lead their thoughts into another channel before the mad fit comes on.

No patient must ever be left unwatched, but they must be unconscious of it, so tactfully and quietly must it be done.

Among every group of patients sitting or walking about are one or two quiet, watchful nurses, making a cheerful spot of colour in their pretty uniforms of pink, with spotlessly white aprons and caps.

Some take their charges for a stroll in the grounds, others flit about the cheery day-rooms and corridors, keeping an eye on those who remain indoors. Others again work busily in the well-ordered infirmary under the supervision of the fully-trained hospital sister, while still others engage their patients in a game at tennis or croquet.

All are treated as mentally-sick, not mad, patients, in the old acceptation of the word, and the nurse must be careful to note all the different phases of their mental condition, on which so much depends for the perfect carrying-out of the medical treatment of the superintendent.

Such work must surely demand intelligence of a higher order than that of the old-time asylum "attendant," who worked under such a different *régime*. And not only is head intelligence needed, but also what may be termed heart intelligence, for the whole work now carried out at Woodilie is founded on the spirit of love; and trained nurses will find there a great field for most important work. In this treatment amusement finds a large place. Weekly dances, concerts, and other entertainments are held, and the magnificent hall in which these take place is said to be the largest in Scotland. A lady pianist is attached to the staff of officers, and is very constantly employed in playing to the patients, both in the evening, during meals, and at intervals through the day in various sitting-rooms. The nurses are not forgotten in the amusement schemes, and have many opportunities for recreation, away from the asylum as well as within the walls. A cycle room is one of the features of their home, and ample "off duty" time is allowed in which they can indulge in this form of exercise, and generally on fine days they can be seen flitting about the roads in the grounds, enjoying a quiet spin.

The matron, who took me over the huge building, has the interest of the work much at heart, and also the welfare of the nurses. She has effected many changes in the nursing staff and in the care of the patients, and is anxious to attract the best class of nurses. She will gladly welcome earnest workers, and will afford them every opportunity for a thorough training in mental nursing.

### OUR CONVALESCENT FUND.

It is with much pleasure that we acknowledge the following contributions: The Nurses of Leigh, Lancashire, 3s; Nurse H., 5s.; Miss F. T., N.B., 2s. 6d.; Miss Louisa J. A., £1 and E. L. W., £1.

### Echoes from the Outside World.

AN OPEN LETTER TO A HOSPITAL NURSE.

THE opinion that the British Government is as strongly averse to war with the Transvaal Republic as the most ardent advocates of peace with honour could desire has been strengthened by the speech of the Duke of Devonshire in Derbyshire. But if the Boers will have war there is no way out; and Mr. J. B. Robinson, who probably knows as much about the Transvaal as any man in England, declares that weeks ago they decided upon it. He also affirms, in effect, that the great obstacle to progress and therefore to peace is ignorance. His description of the Boers cherishing the idea that the Transvaal is the centre of the world, swallowing blindfold all the marvellous statements of their own organs, and satisfied that they can completely overcome any troops we can send against them, really supplies the key to the situation. In such circumstances there is no room for speculation. The Boers want to fight because they object to the British demands; because they have no idea of the British resources; because they are under the delusion that they will beat the enemy out of the field. I notice that a body called the Transvaal Committee of "Liberal Forwards" telegraphed the speech of the Duke of Devonshire on Saturday to Mr. Kruger, assuring him that the Duke's statement that Ministers do not threaten Transvaal independence may be relied upon. This is all very well; but don't you think that if these gentlemen had taken pains to enlighten the Boers as to the real nature of the risks they run in provoking a struggle they would have done their friends a better service?

SIR THOMAS LIPTON is the hero of the hour, and the name of Captain Hogarth is, I find, as much on the lips of the British public as that of Mr. Chamberlain. For, whatever may be the results of the other races between the "Shamrock" and the "Columbia," the splendid manner in which Sir Thomas Lipton's yacht acquitted herself on Tuesday has justified all the confidence which was felt by experts in the capacities of the vessel, and in the seamanship of her commander. This week even the interest in the Transvaal crisis has been put into the shade by the excitement about the opening of the great nautical struggle for the America Cup; while the Venezuela Award, certain to be known hereafter as an epoch-making event, was passed almost unnoticed. As in the case of some of the test cricket matches between England and Australia, there is a reasonable feeling that time alone robbed the British yacht of victory in the first bout.

ON Saturday I went to the special matinée of "King John," having luckily obtained a seat some days before. The vast house was simply packed, chairs being brought in from the vestibules in order to meet the demand for additional stalls. I was much impressed with the difference between the John which I had pictured in my schoolroom days, and John as he is impersonated at Her Majesty's. As a child one is taught to look upon him as a poor weak craven, skilful only in his cunning, and plain and sinister in his appearance. Mr. Tree's John is tall and commanding, his eyes are full of fire, his speech and gestures are dignified and kingly. His defiance of the legate made the submission of the French king seem quite tame and cowardly; in fact, all my sympathy was with John in his bold defiance of the Pope and his decree. Later on in the piece, especially after the death of Queen Elinor, some of King John's calm assurance leaves him, the expression of his features grows more nervous, the loss of his mother robs him of his self-confidence, and his craftiness becomes more apparent. In the scenes with Hubert Mr. Tree is at his best, and when commending Arthur to his charge his by-play is marvellous. He kept his audience spellbound while with his sword he lightly cut the daisies growing at his feet, showing without a word that thus might Hubert deal with the boy, who, like the flowers, was standing in his path. One does not associate Julia Nielson with the *rôle* of a sorrowing mother, but she invested the part with her usual charm and grace without detracting from the necessary sadness, and in her flowing black robes and with her unbound hair she looked the very picture of tragic grief and desolation. I was anxious, by the way, to see how she would respond to the French king's bidding, "bind up your tresses"! She just gathered them into one big coil and twisted it loosely at the back of her neck, without a ribbon or a pin of any sort. I cannot say much for the durability of the coiffure, for later in the scene the frenzy of her despair easily brings her tresses about her shoulders again; but, in these days of ties, and binds, and clips, it must have taken a little practice to learn how to knot up a wealth of hair, even for a few minutes, with no extraneous aid. As to little Arthur, I was especially desirous of seeing Master Sefton in the part, because as a rule it is assigned to a girl, it being impossible to find a boy sufficiently artistic and talented to look and act the character at the same time. Having seen him, I no longer wonder that Mr. Tree was so eager to secure the present representative of the unlucky little prince, for, although fourteen, Charles Sefton does not appear more than eleven at the most, and is a picturesque little person, with a sad childish face. The genuine boyish terror he exhibited when he feared his eyes were going to be put out was wonderfully real, and quite a triumph in its way. The two tableaux which are introduced are very magnificent, but I could not help thinking that they detract somewhat from the artistic setting of the play.

THE first Sunday in October has almost assumed the character of Harvest Festival Sunday in London, because it is so frequently chosen for the yearly thanksgiving for the fruits of the earth. The service I attended was held with strange surroundings, for I went to the chapel attached to the Middlesex County Asylum. It is a large, handsome building, beautifully kept, and on Sunday, decorated with growing pots of flowers, autumn foliage, and piles of ripened fruit, it looked its very best. The flowers were arranged with considerable skill, the grouping around the lectern and on the altar—the latter of apples, white flowers, and ferns—being done most judiciously. At the west end is an alcove, and in this fruit and vegetables were banked up most effectively, the various shades of colour showing up well in contrast against the brick wall. The choir, which is composed of male attendants in uniform on one side, and nurses in their black dresses and snowy, billowy caps on the other, acquitted themselves admirably, one of the younger nurses singing a solo in the anthem with a voice as clear and as high as that of a boy. The patients were there in great numbers, many of them being allowed to attend the festival who are not considered sufficiently well to be regular worshippers. Consequently more had to be taken out than is usually the case. The beauty of the church and the heartiness of the music unduly excited one or two, who commenced to sing in the wrong place or otherwise failed to behave as they should, and had to be removed. Amongst the poor epileptics, who are the most religiously inclined, and who much enjoy their services, the pleasure in a few cases produced a sudden seizure. But so quickly and cleverly were they silenced and carried out that the disturbance was over directly. The two points which impressed me most were the marvellous colour of the ribbons on the women's hats—I believe they are allowed to choose their own, and the majority are very bright—and the clank, clank of the keys hanging from the nurses' and attendants' waists, which knocked against each other as the wearers walked up and down the church.

### The Crisis in the Transvaal.

### NURSING IN THE CAMP AT FORT NAPIER, PIETERMARITZBURG.

By an Alexandra Nurse.

At the present moment the account which a correspondent from the capital of Natal sends us of her work as a nurse in the camp at Fort Napier among the women and children will be read with particular interest. Writing on September 9th, our correspondent says: "The work is undertaken under the auspices of the Soldiers and Sailors' Families Association and a local committee here. I have been engaged in it for sixteen months, and have found much pleasure in it, though the heat is a little trying. Apart from the greater heat of summer we are often visited by very hot winds which are neither pleasant nor bracing."

The camp occupies a splendid position on rising ground at the head of the town, is quite close to the railway station, and commands a magnificent view of the surrounding country, including Table Mountain, Natal, the river, and the valley of the Umsindusi.

A new garrison church was opened last December. This is a great boon to the men, as formerly the heat of the open-air service was very trying. The camp grounds are very extensive, and include a cemetery which is kept in excellent order. The city people are very proud of the camp in their midst, and a good feeling exists between the military men and the civilians.

Government House, the residence of the Hon. Sir J. Hely-Hutchison, is just outside the camp gates, and within ten minutes' walk of the centre of the town. The shops and stores are very good. The population is mixed—there are many Kaffirs, Coolies, and Arabs. The Kaffir boys are useful as house servants or washing boys. The Coolies and Arabs seem to do a brisk trade as they go round the camp daily.

At present we have over 104 women and about 200 children in the married quarters, but as there is not accommodation for all of these in camp quite a number are lodged outside in the town. There is no female hospital in the camp, hence the women and children are nursed in their own homes. Confinement cases are attended by a nurse from town, or one of our sergeant's wives, who holds a certificate, and is very good at her work. Infectious or very serious cases of illness are sent to the Government or City Hospital at the other end of the town, the accommodation in the homes of the patients not being sufficient for the proper nursing of these. I nurse under a surgeon-major, whose services are much appreciated by the women, and I also receive a warm welcome from them and expressions of gratitude for services rendered. Apart from the help given in sickness, they often consult me, and seem glad to be advised regarding the better ordering of their home, or even as to the best way of laying-out their hard-earned money; many of them take a great pride in their homes and children, so that I am pleased to say they would compare favourably with the homes in town of a better social status. This is by no means a cheap place in which to live at any period, and now that the talk is all of war in the Transvaal, a number of things have gone up in price.

During the last few weeks we have had an epidemic of measles; forty little girl patients were down at the same time, but all are making good recovery. Since my term of service began we have only had two deaths. One was that of a little girl of nine months from natural disease, the other a little girl four and a-half years from shock, the result of a burning accident. Last year we had many cases of chicken pox, pneumonia, whooping-cough, &c., but all were spared to be, I trust, a source of joy and comfort to their parents, a credit to their regiment, and mayhap the future defenders of our country, or the true helpmeet of our brave soldier lads.

### The Heaven-Born Doctor.

From Life.

When he comes into the room you feel all at once that he brings with him an atmosphere of strength and power. You feel stronger yourself because he is there, and your patient opens tired eyes and smiles with a renewal of hope and courage, as that strong personality makes itself felt.

He is never gloomy nor depressed. If he is anxious, he skilfully hides the fact, and his smile is as beaming and kindly for the patient who is desperately ill, as for the one who verges on recovery. The very sunshine of his smile carries hope with it; the sick person wants to get well when he meets the glance of those kindly eyes. The deep voice is as cheery and helpful as the smiling eyes.

"Well, your majesty!" he says cheerily, and the patient, who a few minutes before felt herself in the lowest depths of misery, feels now a glow of warmth and brightness.

There is nothing stereotyped about him; no bedside manner, no tiresome hushed voice, which makes you think you are really going to die this time! No! he is natural, normal, healthy; his voice is well modulated, his step is firm, his touch is gentle to tenderness. Yet all the time he is just himself, with no alterations carefully adjusted for the sick, which are so annoying to the patient.

Nothing escapes those keen eyes. A moment or two beside the bed and he has diagnosed the matter. He has that wonderful instinctive power of diagnosis, so rare as to be indeed regarded as a gift from Heaven. He puts his finger, as it were, on the right spot at once.

But he is never hurried; no taking out of a large watch and a scrambling departure. There is no scamping of details here; no "Well, nurse, I'll leave that to you"; no shrug of the shoulder outside the patient's door, and "Well, there's nothing to be done; we must just let things take their course."

Be a case chronic, or be it acute, this doctor does *not* let things take their course without doing his level best to direct that course. He considers every tiniest detail that makes for the patient's comfort, whether those details are of vital and essential importance or not. He goes carefully into the question of diet; he reads and grasps the nurse's written report; nothing in the interest of the patient is too small or too insignificant for his interest. Has the sick person a little ache or pain? He tries by every means in his power to alleviate it. He does not say "Oh, it is part of the illness, and we cannot help it." He will at least alleviate it, if it cannot be cured.

His personal magnetism is so great that those who come in contact with him do whatever he wishes from sheer love of the man, and desire to please him. It seems well worth while to earn his hearty thanks, to call forth that sunny smile, and it is not pleasant to incur his anger or to feel that you have vexed or grieved him. Not that he ever rages at you, or loses his temper, but a quiet word of disapproval from him is far worse than a storm of temper from another doctor.

He is so courteous to his nurses, he never treats them as though they were tiresome encumbrances, or as the dirt beneath his feet. He is as polite and civil to them as if they were duchesses. He has no fear of compromising his dignity by being polite to his subordinates. One realises as one watches him the truth of the poet's words,

"The greater man, the greater courtesy."

In hospital it is the same. His out-patient rounds are like a royal progress, as one of his own students has said. The poor men and women he is treating, come, like everyone else, under the spell of his personality, and want to do exactly as he wishes. In the wards, as in private houses, his going leaves a blank, his coming brings strength and brightness, for to alter slightly the words of our great poet,

"He comes, and the sunshine comes along with him."

### A Model Lodging-House for Nurses.

By a Travelled Nurse.

It was in New York that I came across the model lodging-house for nurses, which I should like to describe for the benefit of those who are interested in the ways of life of their nursing sisters abroad. We have learned to associate the term "model lodging-house" with charity and "assisted effort." So I hasten to remark that the model lodging-house in question bore no suggestion of charity or of being under philanthropic auspices. It was a lodging-house for nurses of high professional standing, but on a somewhat different footing from the average house of that type. It may be necessary to remind those who have never been in the States that the high price of labour and servants' wages prevents the adoption of the English method of lodgings, save by those who have more money to spend than the average private nurse. The compromise of the boarding-house is the alternative, since lodgings with meals served in one's own room are a very expensive luxury in the States.

Now there are many nurses who do not like living in a boarding-house. After the wear and tear of private nursing it is a relief to come back to one's own quiet room. The form, ceremony, and publicity of a boarding-house are by no means soothing to the tired worker. Some New York private nurses have therefore adopted the American system of "light housekeeping" in preference to living in a boarding house. Light housekeeping means the taking of a bed-sitting room, which contains a little stove whereon light cookery may be done. The grilling of a chop, the cooking of fish and small dishes enables the nurse to prepare light meals for herself between cases. And she can supplement these, if she so desire, by taking an occasional "square meal" at a restaurant. The landlady who keeps a house for the accommodation of "light housekeepers" provides no service for her lodgers. A nurse, therefore, who selects this method of living—very common in America—must "do" her own room, sweep, dust, light her own stove, carry down ashes and refuse, &c. To English ears this sounds a very uncomfortable way of life, but American women brought up under domestic conditions, so very different from ours, regard "light housekeeping" as extremely satisfactory and comfortable. Under this system the nurse can choose her own food, as she cannot in a boarding house, and pays only for what she really consumes. The room is kept for her while she is at her cases. A small underneath cupboard holds her stock of coal, a little pantry contains her store of food—for entire buildings are specially constructed for a light housekeeping *clientèle*—and each separate room contains complete accommodation for coal and a due cellar equipment.

Nobody can say that it is hygienic to live and sleep in the room which holds your provender, clothes, coal, and stove. But the price of living in New York settles the question, and the individual who is not burdened with superfluous dollars speedily adapts herself to light housekeeping conditions, and manages to make her life pleasant enough.

The model lodging-house I speak of is on a more wholesome system than the light housekeeping plan. While it provides bed-sitting rooms for nurses it does away with the necessity of cooking stoves in the separate rooms. A delightful little kitchen is at the disposal of nurse lodgers, and here each nurse cooks her own food when and how she likes. All requisites are provided, thus doing away with the necessity entailed by "light housekeeping" of each nurse providing her own kitchen kit. The stove is always ready, and for the privilege of kitchen and coals a certain weekly or daily sum is paid. Every nurse using the kitchen is expected to leave things as she found them—to wash the saucepan, plates, and dishes she has used, and neatly put them ready for the next comer. Two or three nurses sometimes club together for a chicken or some other dish too large and costly for one. And under such circumstances the purchasers may choose to dine in company, rather than to divide the dish into separate shares. Nice sociable little meals are accordingly arranged on co-operative plan. Clubbing together has other points to recommend it, since one of the two or of the trio of co-operators can serve as cook—for too many cooks might spoil the dish. By taking the post of cook in turns the labours of the kitchen are happily divided, so that all the time between cases is not swallowed up in the performance of domestic duties. If two nurses share a room the expenses of the model lodging-house prove extremely moderate. And this is a practical method of keeping down expenditure, and in reality allows a good deal of privacy, for the two nurses will rarely be "in" at the same time. I could not help thinking, as I stood in the busy kitchen watching the cheerful frying of oysters and flannel cakes, the baking of potatoes, and the manufacture of sundry pies, that such little culinary duties had the effect of "keeping the hand in" for that sick cookery which is so essential to the private nurse. And my mind flew back to the meals I have seen provided for English private nurses quartered in economical lodgings. I meditated on the greasy chops brought in by untidy maids-of-all-work on plates by no means above suspicion, the spotty table-cloths, and general want of daintiness of inexpensive lodgings in London. By contrast there is no question that the nurses of the model lodging-house in New York, who turn out their appetising and cleanly-served little meals on bright dishes washed by their own hands, have a much more tempting commissariat than that of the nurse who depends for her diet upon the culinary talents of a London landlady.

### A Youthful Advocate of Temperance.

In a large, airy ward in a children's hospital stood twenty beds, each with its tiny occupant. Ten minutes earlier there had been one empty bed, but now a restless little child lay in it. I saw on taking her card from the porter that she was on the danger list, and thought, as I anxiously watched, that her time on earth was short, and that she would soon join the angels of whom she talked. But God had some work for this little one to do, and she slowly struggled back to life. During the first few days, when the fever ran high, her one cry was for ginger beer. "Chinger beer," she called it, and when the stimulant which had been ordered by the doctor was given her, she pushed it away, saying, "Wicked nurse, to want baby to drink spirits. No spirits in heaven, only chinger beer and beef tea. Of course," she added, after thinking, "only spirits with wings, not drinking spirits." She liked the blinds drawn up at the peep of day; "I want to see the angels shut their eyes," she would say, meaning the stars. When evening came she used to put her tiny hands together, saying, "Please Dord, make baby better, baby's welly welly poorly and tired, and make Nurse throw all the drinking spirits away." "When I get big," she would say, "I shall go out of doors and ask naughty men and women only to drink chinger beer and beef tea."

### To Nurses.

In order to increase and vary the interest in the *Mirror*, we invite contributions from any of our readers in the form of either an article, a paragraph, or information, and will pay a minimum of 5s. for each contribution. All rejected manuscripts are returned in due course, and all payments for manuscripts used are made at the beginning of each quarter, *i.e.*, January 1st, April 1st, July 1st, and October 1st.

### Novelties for Nurses.

### AN EFFECTIVE BOOT TREE.

The accompanying illustration shows a most simple, effective, and cheap boot tree, to be had from the London Shoe Company, 123, Queen Victoria Street, E.C., and 116-117, New Bond Street, W. Everyone is alive to the advantage of keeping boots and shoes in good condition, and thus increasing their durability, by the use of trees. But hitherto trees have been expensive articles. The ingenious "Shooco" tree sold for 1s. 8d. by the London Shoe Company enables everyone to secure these really necessary additions to the wardrobe. They are strong and well made.

### SHOES FOR NURSES AT GOOCH'S STORES.

There are few windows in the Brompton Road that appeal so irresistibly to the fancy of the passer-by as those which extend from 67 to 77, known as Gooch's Stores. The most fascinating one of the number, perhaps, is that devoted to boots and shoes. From the daintily-embroidered, high-heeled Louis XV. to the sturdy, serviceable moorland brogue there is an infinity both of gradation and design. Nurses who have much standing, and are more or less continually on their legs, will do well to avoid the very smart, pointed-toe models, as, however charming they may be to look at, they are weariness and vexation of the flesh to wear. The total, and, alas, not infrequent breakdown of a nurse's feet is due more often than not to her desire to look smart, without considering the result. A high heel throws the foot forward and broadens it, besides numberless other evils, and is always more or less fatiguing to wear for long at a time. We therefore most earnestly counsel those who value their health and wish to pursue their calling in ease and comfort to avoid all extremes in the way of narrow toes and high heels. A broad sole and square, military heels are undoubtedly the proper wear for nurses and others who have much walking or standing to do. Recognising this principle, we are glad to see that the enterprising manager of Gooch's Stores, whose experience extends over many years, has recently designed an excellent shoe which has the merit of not only being exceedingly comfortable but is neat enough in appearance to please the most fastidious. This shape, known as the "Samaritan Ward Shoe," is made in delightfully soft glacé kid, with sensible square heels about an inch in height and capped at one side with india-rubber to render them noiseless. The instep is supported by an ingenious contrivance to which we call special attention, which is placed between the outer leather and the lining, thus reducing the chances of flat foot to a minimum, while supporting feet already weak. Straps cross and button over the instep, thus giving it additional support. We were surprised to hear that the price was only 12s. 9d., very moderate indeed for such a high-class article and one that is guaranteed to survive at least half a dozen pairs in a cheaper variety. In conclusion, we strongly recommend our readers to pay a visit to 67, Brompton Road, and see for themselves, for seeing is believing, and believing in this case would most certainly end in purchase. There are also other novelties in the boot and shoe line which merit inspection, and about which we shall have something to say on a future occasion.

### AN EXCELLENT HAIR TONIC.

The most refreshing and excellent hair tonic we know is that sold at the 4,711 Eau de Cologne Depot at 62, New Bond Street under the name of Captol. It is manufactured according to the directions of Dr. Eichloff, of Elberfeld, in Germany, and has undoubted invigorating properties. It is most refreshing and is pleasantly perfumed, and has no greasy effect on the hair; indeed, it adds greatly to its appearance. It keeps the skin of the head in excellent condition.

### DAINTY NOVELTIES FOR THE SICKROOM.

Everyone knows the difficulty of finding suitable presents for the sick, and especially for those chronically ill. A recent visit to Messrs. Hemmings, 28, Conduit Street, proved to us that with their assistance a useful and ornamental addition to the sickroom could be found at once. In fact, it was almost a matter of *embaras de richesse*. It is not easy to describe dainty trifles graphically, and so we have given illustrations of a few of the charming novelties that struck our fancy. First there is a delightful beef-tea set, so dainty and elegant that it could not fail to render the tedious beef-tea more interesting and appetising than when served in the usual manner. The stand is silver or plate, the bowl of white china or of the blue Worcester willow pattern, whichever the purchaser prefers. The little toast rack, and pepper and salt pots, make the little beef-tea service as complete and compact as possible. A simpler and still less expensive set can be used for bread and milk, gruel, or soup equally well, as it consists of the bowl on a silver stand with spoon in addition only, as shown in the drawing. Next we would mention the contrivances to make the dark night less lonesome and dreary in sickness. Charming little shaded night lights are made of silver without any ornamentation for this purpose. The night light slips into a cup just large enough to hold it; on one side is the shade to keep the direct light from the invalid, whilst its polished surface diffuses the light over the room. All invalids do not like a continuous light in their room, but they are always desirous of a light close at hand. For such the neatest of little contrivances is supplied. This consists of a matchbox upon which is fixed a small candle and extinguisher, so that a light can be procured at a moment's notice, and as quickly extinguished. The silver matchbox with luminous tablet to reveal its whereabouts will form a welcome present also. Matchboxes of a particular kind like this are very desirable in a sickroom, as they are much less apt to stray from their accustomed place than the ordinary boxes which appear to be everybody's property. Means of learning the time at night is provided by the use of that most fascinating of time-pieces, an electric light clock. As shown in the illustration, they are cased in leather with electric battery, so that by pressing a button on the top of the clock, a tiny electric light is illuminated above the face of the clock, which reveals the time. The electric charge lasts a long time, and can be easily replenished. This clock is provided by Messrs. Hemming at a remarkably low price; indeed, all their goods are most excellent in quality and as reasonable as possible in price. I cannot close this notice of their many useful specialities without mentioning their nice little medicine spoon, the handle of which is practically a silver straw, through which medicines can be administered which are apt to discolour the teeth. Messrs. Hemming are glad to send illustrated catalogues on application, or show visitors their varied collection of productions.

### For Reading to the Sick.

"My son, give Me thine heart."—*Prov.* xxiii., 26.

He chose this path for thee.
No feeble chance, nor hard, relentless fate,
But love, His love, hath placed thy footsteps here.
He knew the way was rough and desolate;
Knew how thy heart would often sink with fear;
Yet tenderly He whispered, "Child, I see
This path is best for thee."

He chose this path for thee,
Though well He knew sharp thorns would pierce thy feet;
Knew how the brambles would obstruct thy way;
Knew all the hidden dangers thou would'st meet;
Knew how thy faith would falter day by day;
And still the whisper echoed, "Yes, I see
This path is best for thee."

He chose this path for thee.
What would'st thou more? This sweeter truth to know,
That all along these strange, bewildering ways,
O'er rocky steeps and where dark rivers flow,
His loving arms will bear thee "all the days";
A few steps more, and thou thyself shalt see
This path is best for thee." —*Anon.*

Jesus calls you! Daily, hourly, His gentle voice is pleading, amid the throng and in the silence, waiting until you pause, and answer. How can I pause, much less answer, when, in the short, hurrying day, I am beset with trials? Duties follow in such quick succession that I have not time amid the "coming and going" for brief reflection even. But have you ever tried, when the strain is greatest, to lift up your heart for one short moment in prayer? Ah! then above the tumult without and the unrest within, the impatience, the weariness, the discontent, perhaps, to which at times the best of us are prone, you will hear the Master's voice in low, penetrating tones, saying, "Follow Me, and thou shalt find peace for thy present distress, and strength for to-morrow's task. Amid the tumult I am with thee. I know thy difficulties, thy weakness, and thy pain. Come unto Me, leave all, and thou shalt be at rest." Yes! dear Lord, I fain would come, but must I leave *all*, and follow Thee? The cross, which though it presses heavily, I yet must carry after Thee; the sorrows and the sufferings of those who I am bound to love and help, for Thy dear sake; the weary days and sleepless nights, must I these forego since 'tis Thy will? Dear child, I do not ask you to leave your cross; I ask you to take it up daily and to follow Me; to leave at the foot of My Cross all those thorns in the flesh of self-dissatisfaction, impatience, spirit-weariness. Then, rising up soul-strengthened by their discardment, to go forth. "My presence shall go with thee, and I will give thee rest." Dear Lord! I will arise and follow Thee, into the sunshine if Thou wilt, to the shadows of Gethsemane if Thou so decree only,

"Take Thou my hand,
And through the wild
Lead gently home
Thy trusting child." —*Julian.*

It is possible even amid the the claims of business to arise at once and follow Christ. While the day is ours let us go forth to minister in sickness and in health, by acts of self-denial, patience, and gentleness, to those among whom God has placed us. Treading in His steps, following where He leads, may we at last hear His loved voice saying, "Well done, good and faithful servant; enter thou into the joy of thy Lord."—*F. B.*

Is this the peace of God, this strange, sweet calm?
The weary day is at its zenith still;
Yet 'tis as if, beside some cool clear rill,
Through shadowy stillness rose an evening psalm;
All the noise of life were hushed away,
And tranquil gladness reigned with gently soothing sway.

It is not that I feel less weak, but Thou
Wilt be my strength; it is not that I see
Less sin, but there is pardoning love with Thee,
And all-sufficient grace. Enough! and now
I do not think or pray; I only rest
And feel that Thou art near, and know that I am blest.
—*Anon.*

### Everybody's Opinion.

[Correspondence on all subjects is invited, but we cannot in any way be responsible for the opinions expressed by our correspondents. No communication can be entertained if the name and address of the correspondent is not given, as a guarantee of good faith but not necessarily for publication, or unless one side of the paper only is written on.]

### NURSES' READING SOCIETY.

L. G. Moberly writes: I am sorry that in setting books for next quarter I have been unable to set one upon aseptic surgery, as I had hoped to do. But the book that I want to set is not yet published! It shall be set later on. May I once again remind nurses that the address of the Lending Library is Mr. Glaisher, Wigmore Street, Cavendish Square, W., and that all arrangements about the library must be made with him—not with me. It would save nurses stamps and the time and trouble if they would kindly understand that unless I state any rules upon their examination papers, there are no rules. The papers simply have to be answered and sent in to me on or before December 8th.

### CATHOLIC LETTER GUILD.

The "Honorary Secretary" writes: May I call the attention of Roman Catholic nurses to the above guild? It is well-known that those immersed in their work, and having few outside influences, often long for some means of being brought into communication with others with whom they may be in sympathy. A nurse has often real need of "getting out of herself," as it is called, and amongst other instances my attention has lately been directed to one in the provinces, begging for correspondence with a lady in London to whom she might write on anything except nursing. Those who would find a friendly letter a pleasure and a help can obtain further information by writing to Miss Denham, 61, Carey Street, Lincoln's Inn, London.

### A MATRON'S DUTY IN REFERENCE TO RULES.

"A Late Lady Superintendent" writes: If a thoroughly trained hospital matron be given by a committee, on her appointment, a set of rules to be followed, and afterwards finding by hearsay, or by their action, that that committee (mostly through ignorance of hospital organization or because they wish to see no fault in their own institution) evidently do not wish her to use the power given her in those rules so as to maintain right conduct, discipline, and training in nursing in the hospital, is she justified, for the sake of policy, in letting things be, and simply acting as a figure-head, receiving a salary, but laying aside the traditions and training of her own nursing school and allowing things to go on which she knows to be wrong? In that case, to take the lowest ground, is she not damaging her own reputation as a matron by continuing in such a position? There is also the question of faithfully carrying out the rules given to her, and to the best of her ability serving her committee according to the training and experience she has had, and owing to which she was appointed. I should like to hear what others have to say on the subject.

### ONE OF THE FAMILY.

"M. S." writes: After all the discontented letters lately appearing in the "Nursing Mirror" about the treatment of private nurses, "Vera's" and "Beth's" letters come like a delightful sedative; they contain all the good sense which is so essential for nurses to possess, and which makes them such helps in sickness and sorrow. As I am only a would-be nurse who recently failed at exams. in one of our great training schools, my opinion, perhaps, is not worth much; but I am at present helping to nurse a sad chronic case, and know something of the consideration and care due to the sick and their relatives. I have lived in good families for years, and have had the opportunity of forming a fairly true opinion of their general idea of trained nurses. "Vera's" opinion is, I believe, correct. It is "not always convenient or possible to treat a nurse as one of the family," though I am certain that all well-bred people desire to make her stay with them (let it be remembered always a time of trouble and anxiety) as agreeable as possible. As regards meals with the servants, I fully sympathise with nurses who are gentlewomen by birth; they must feel rather out of place in the kitchen, but I cannot agree with the one who thinks the servants' hall the only place where scandal and gossip is indulged in. If it is not convenient for the nurses' meals to be taken to the library or into the dining-room after the family has left it, there is frequently in a large house a dressing-room, or some room leading out of the sickroom which allows of ventilation, where the meals could be served, and then the nurse could make the atmosphere as pure as she likes. In small houses where there are only few conveniences, a nurse's training ought to help her to make her surroundings as healthy and pleasant as is necessary, and to make the best of existing circumstances. If she has "Beth's" sunny temperament it won't be difficult either, for if a nurse possesses true sympathy with her patients and their friends, she will find her pleasure in duty well done, even at the cost of a few creature comforts, and the less a nurse thinks of herself, the more she will be thought about by others.

"A Nurse from the West" writes: The thought struck me whilst reading "Vera's" opinion about nurses being treated as one of the family, that probably "Vera" would feel differently if she were placed in a nurse's position. A nurse naturally feels lonely on entering a house as a perfect stranger. She will probably be an inmate for a few weeks only, would it not therefore be kinder to try to make her as happy and comfortable as possible, and to make her feel at home the short time she is off duty? I presume her patient is a member of the family, therefore if she can take her meals with her patient in the bedroom, why not sometimes with the rest of the household? Because the nurse is "a stranger in a household" is the ve y reason why she should be treated with kindness and consideration. Nurses are but human—they are not perfect, though some people expect them to be so, and they sometimes adapt themselves to most trying circumstances. If "Vera" has met with some nurses who do not come up to her ideal, doubtless, on the other hand, there are many who are disappointed in the families into which they enter.

### NURSING IN FEVER HOSPITALS.

"A Late Charge Nurse" writes: Having read the papers in your columns on "Training in Fever Hospitals," I should like to say a few words. Last October I went as charge nurse of a scarlet fever ward to one of the hospitals under the Metropolitan Asylums Board. I went with every intention of staying, but left at the end of seven months. The nurses' quarters were almost perfect. Shortly after my arrival a new block was opened for the accommodation of the charge nurses and night staff, containing, besides bed and bathrooms, &c., dining-room, sitting-rooms, and library—fine large rooms, comfortably furnished. The food was good, but badly cooked and served. I had a pretty new ward and many delightful little patients. After a busy general ward I found the work a little tedious, but I soon got very interested in my new patients. The matron and medical superintendent were always very considerate. The great drawback I found to be the assistant nurses. Their particular grievance seems to be that they have a trained charge nurse over them who draws a bigger salary than they do, and who leaves them to do the work. For my part, I found the assistant nurses, with a few exceptions, extremely lazy, and objecting very much to be taught. I believe that when an assistant nurse was left in charge of the ward that she did her utmost, but, as a rule, as soon as the charge nurse crossed the threshold all her interest seemed to go. I certainly learnt in my fever experience that if I wanted a thing done properly I must do it myself, or, at the least, see it done. Fortunately I always had a sort of liking for what is termed menial work, for my assistants thought it quite beneath their dignity to keep the ward tidy and clean. I have many times seen charge nurses sweeping up the crumbs while their assistant nurses, who were supposed to have swept the ward, were away changing their aprons.

"Credenda" writes: I read a charge nurse's letter with no little interest, and I feel that I should like an answer to a simple question or two. How is it that so many of the poor, harassed charge nurses can reconcile their consciences to allowing the "untrained, lethargic assistants" to do all the treatment in the ward, as well as the menial work? One would naturally think that on finding the "untrained" assistant not up to her standard the charge nurse would prefer to do all the dressings and treatment herself rather than allow a nurse to do it whom she considers incompetent. I have worked under the Metropolitan Asylums Board now for nearly three years, and during that period I have not met with more than one charge nurse who did not consider that she had performed the whole of her duty in the way of treatment when she had taken the patients' temperatures. We do not profess to be adepts at general nursing, but I think we can give general trained nurses a good start and beat them pretty thoroughly in the matter of syringing throats, giving hypodermics, and nasal feeds. Indeed, not infrequently the "untrained" assistant has had to show the competent and fully-trained nurse how to give the latter. I can hardly think "A Charge Nurse" has worked under the Metropolitan Asylums Board. Had she done so she would surely be aware of the fact that in several of the board hospitals first assistant nurses are always placed in charge of the wards on night duty. This plan has been working for over three years, and has been found very satisfactory. I have always understood that charge nurses are in the wards to be an example to the assistant nurses and to see that the work is thoroughly done. But can we respect a woman who deliberately takes two hours in the morning for dressing instead of the three-quarters of an hour which is her due? Can that nurse superintend the work of the ward and be in her room at the same time? I fear not; yet the majority of the charge nurses in the hospital to which I am attached regularly leave their wards for an hour and a half to two hours every morning. Is it then any wonder that instead of respect we feel only tolerant contempt for the charge nurse who in nine cases out of ten is merely a figure-head? When the Board of Management at Norfolk House define what the charge nurse's duties are, more explicitly than is done in the present rule book, then, and then only, will there be any satisfaction felt by the assistant nurses. Of one thing we feel very sure, and that is that the public do not desire to pay a nurse £36 a year to sit in a ward doing next to nothing.

### PRIVATE NURSING.

"NURSE MARY" writes: May I say something? I have had five years' experience of nursing in private. During the time I have received nothing but kindness and consideration from the family and friends of the patient. I have been treated with the greatest respect, and found that "nurse's" wants were among the very first to be attended to. So thoughtful and kind have people been that I have many times been simply astonished, and I have felt equally grateful. And if one cannot in all cases have every comfort, I find that the members of the household themselves usually feel it intensely if they cannot give one all the comforts one might wish for. This alone helps one to bear discomforts. I have always been treated quite as one of the family, and had daintily served and cooked meals—sometimes alone, sometimes with the family. In one house the lady brought me a cup of tea every morning at half-past seven that she had made herself. Is not this thoughtfulness and kindliness? One likes to meet as friends and fellow-workers the people one may come in contact with, and leave them feeling that each have benefited by knowing one another. I could not go in a house in a spirit of criticism. Meet each other half-way, and help to smooth, not make, difficulties, I say. I am partially trained, only. I could not resist just writing this.

"A NURSE FROM WORCESTERSHIRE" writes: I have read with interest the different letters anent the woes of private nurses, and would like to give my experiences. I have been private nursing, attached to an institution, nearly five years, and on the whole am fairly happy in my work, which I love, though I am obliged to do something for my living. It seems to me that the most difficult matter to deal with is the position we should hold in the many houses we enter. There can be no set rules as to what we should or should not do; we must be guided by circumstances. For instance, where there are three or four servants it is unnecessary to dust and do such like work, and we certainly have a right to expect our meals served properly, and also comfortable apartments. But in some houses the people are so wretchedly helpless that we cannot help doing many things unprofessional, even to cooking the meals and helping to keep the house in order, though I find that "ladies" sometimes consider this unfits us for nursing them. When a trained nurse has won her position by hard work and close study, it does seem a little hard that she should be treated so coldly and left to live the life of isolation as is often the case. I have sometimes been driving out with my patient, who would talk cheerfully and be glad of my company until someone else has joined us. Then I was completely ignored, without even the slight introduction, "This is my nurse." When visitors call I always leave the room for a time, in order that any conversation may be carried on, and it is surely very little to expect that I should be spoken to pleasantly as they come in and go out. Men usually are much more courteous than women. They evidently have a higher opinion of us, and for that reason many of us may be forgiven for preferring to nurse the male sex. By the time a nurse has gone through the necessary discipline in training she is far too sensible to expect the attention generally accorded to young ladies of society, but she has every bit as much right to be treated courteously. But this has been only one side of my experience. I have made several good friends since entering the nursing world, who help to preserve my enthusiasm and give strength for the trials with the more uncharitable of my sex.

### ON HAPPINESS IN WORKHOUSE NURSING.

"E. R. W." writes: I was very glad to see the picture which your correspondent drew of some of the happiness to be derived in workhouse nursing. I, too, could draw many a happy picture of my workhouse nursing experience, such as the yearly treats, the concerts in the board-room, the magic lantern entertainments by one of the doctors, the volunteer band which came and played under the verandah, and responded again and again, first to one old granny and then to another as they begged "let's have another." But I also see a picture of a girl, aged 22, paralysed, her knees drawn up, sometimes giving her excruciating pain. She was turned out of the hospital after seventeen months' trial as "incurable." She arrived with a flushed, anxious face, clinging to her hospital nurse, dreading the infirmary and the workhouse nurses. The big wards were full; she was put into a "side ward." This "side ward" became the ray of sunshine of the place, for everybody went in to visit "Fanny," and her room was always decked with flowers, and Fanny herself the brightest of them all. Always full of fun, always inquiring how others were; always embroidering, knitting, sewing something for others. She had had a "sweetheart," who, though knowing the hopelessness of her condition, would not give her up. He had died suddenly the previous Christmas. This girl required real, skilled nursing, and to me it seemed worth while sacrificing one's professional standing to bring comfort to such as her, even though one became only a "workhouse nurse"! I could also draw pictures of young men crippled for life, and of dear old aged men and women who had finally come to the workhouse infirmary through no fault of their own. Happiness in nursing is not to be found in "splendid pneumonias," "exciting typhoids," nor yet in witnessing "daring surgical ops." In my case, the drawback to workhouse nursing was not the workhouse master, for he was charming, and where there is no "resident," I cannot help seeing that he should be the right man to keep order, especially where male patients are concerned. But for a lady to leave her hospital where the matron is a gentlewoman, where the whole tone and management of the place is in the hands of clever, cultured people, to find herself stranded under a superintendent who goes out bicycling with the cook and baker of the establishment, and where the progress, organisation, and management of the place is dependent on the opinion of a majority of absolutely ignorant, illiterate guardians, working on an equality with the nurse who is neither educated nor trained, is, I think, more than trying. If only more ladies would take up the work I am sure they would appreciate it.

### The Ethics of Nursing.

### PURITY.

A LAY woman of my acquaintance told me that she one day remonstrated with a nurse for telling professional stories which bordered, and more than bordered, upon coarseness and immodesty. The nurse replied, "Oh, but all nurses lose their modesty and get hardened." "Not at all," my friend replied; "look at So-and-so" (naming another nurse), "you never hear anything professional or coarse from her."

To say that nurses must necessarily lose their modesty is not only a terrible accusation, but it is far too sweeping. Yet is there not sometimes a real danger of our becoming hardened, coarsened, too much accustomed to things and conversation which would once upon a time have shocked and horrified us? It is, to my mind, inexcusable that a nurse should prattle of professional things to the laity, and one would like to feel that no self-respecting decent-minded nurse would do so; but I fear it is a temptation to which many are only too prone to succumb. It is so unpardonable a fault that I do not propose to deal with it at length here. The root of the matter I believe to be this. Nurses live in a very circumscribed world; their work in hospital is to a certain extent a squirrel's round, and naturally professional subjects are the main interests of their lives. The professional is so entirely familiar to them, that when a nurse leaves her hospital and is exclusively among lay people she is apt to forget that the professional is no longer familiar to those around her. One cannot help hoping that it is this forgetfulness which leads nurses to talk so appallingly as they often do of the things of their profession. And they do this not only indoors, but in the streets, on the tops of omnibuses, and in trains. A friend of mine told me once that it made her feel positively sick and ashamed to hear the conversation of two nurses sitting behind her on an omnibus.

Oh! the pity of it! To bring your profession—your grand nursing profession—into disrepute, because you cannot govern your tongue and your mind. Is not that, after all, the root of the matter—the guarding of our minds? If, when you are in hospital, you guard the portals of your mind against all that is coarse, impure, immodest, your tongue will not go astray, either within the walls of your hospital or outside them. Is it not our own fault if we absorb the worst and coarsest side of our work? Is it not in our own power to keep clean hands and a pure heart? And no professions are more in need of clean hands and pure hearts than the medical and the nursing professions.

Was it not Ruskin who said, "You can no more filter your mind into purity than you can compress it into calmness; you must keep it pure if you would have it pure, and throw no stones into it if you would have it quiet"?

How true! "You must keep it pure if you would have it pure." And how infinitely harder to cleanse a stream once fouled at its source than to keep its source clear. Don't let anything enter your mind which can spoil its purity. Your "strength will be as the strength of ten, because your heart is pure." In the great legend of the Grail, who was it who saw the Holy Cup? Parsifal, the pure fool—not the energetic knights with their worldly wisdom, but the simple boy who was pure. Too often hospital talk degenerates into low professional gossip, and one hears things talked about and laughed over which ought not to be chattered about at all.

"Unto the pure all things are pure." Yes; and nothing in a nurse's work need be lowering or injurious if it is undertaken in a right spirit. I repeat and I maintain that it is your own fault if you do not come out of your hospital life a pure-minded, modest, womanly woman.

Keep your mind assiduously fixed on the higher aspects of your work, not on the lower ones. Let the unpleasant details which must be done be also done with as soon as they are finished, and put them out of your mind, and certainly out of your conversation. Fill your mind as much as you can with beautiful and ennobling thoughts; never dwell upon anything which you feel could for a second coarsen you or anyone else. And it is important to remember in this connexion that what may be harmless to you may be bitterly harmful to another; you cannot guard your words too carefully.

But begin at the right end—with your thoughts. Who was it that said, "Guard well your thoughts, for thoughts are heard in heaven." Surely few mottoes could be better or more appropriate for a nurse than those beautiful words, "Wearing the white flower of a blameless life." Cannot we all wear that white flower? And looking round for a quotation to bear out my point, is there anywhere a better one to be found than the old, old teaching of the wise Apostle, "Whatsoever things are true, whatsoever things are honest, whatsoever things are just, whatsoever things are pure, whatsoever things are lovely, whatsoever things are of good report, think on these things."

---

### "Daddy Nine."

THE nurses called him "Daddy Nine" because he occupied No. 9 bed, and all the old men in the medical ward of the L—— Hospital were spoken of as "Daddies" by the nurses. He was admitted with acute laryngitis, and the doctors shook their heads over him, for he was very bad indeed, and utterly refused to have tracheotomy performed. Three days came and went; three very long days for Daddy Nine, for he was suffering very much, and each day found him weaker and his breathing more difficult.

It was the evening of the third day that, as his nurse made him comfortable for the night, he touched her on the arm. "Tell the doctors they can do it," he whispered. So that evening they performed tracheotomy, and Daddy Nine had a screen all night round his bed and a bronchitis kettle. It was noticed that he never murmured, although the silver tube hurt him, and he never closed his eyes all night. It was only when the morning came that he whispered to one of the nurses, "I wish I was dead and buried and done for."

On visiting days no one came to see Daddy Nine. He explained that he had not a friend in the world. "There was Maria and the kid, but they cleared off and left me years back. I a'int set sight on 'em since," he confided to his nurse.

One day No. 10 had several friends to see him, and an anæmic-looking girl with a bunch of pansies presented some to No. 10 and then came shyly over and laid the rest on Daddy Nine's counterpane. He thanked her with his eyes and a nod. Afterwards one of the nurses saw him trying to press the flowers between the pages of the *Globe*.

Daddy died in the surgical ward a few weeks later after a severe operation. The doctors had discovered he was suffering from cancer of the breast, and although for several days he got on very successfully, he afterwards grew very weak, and there was failure of the heart's action. The last thing he did just a few hours before he died was to hand his nurse a small paper packet. She opened it, and a few withered pansies fell out. "Burn them," whispered Daddy, "she'll come next Wednesday an' I shan't be 'ere. She'll never know, but I knowed when I see 'er again as it was Maria's kid growed up." "Poor man," said the nurse, as she folded his hands and closed the eyes of Daddy Nine, "he wandered very much at the last and I'm sure I do not know what it was all about, but I may as well burn the flowers."

### Typical Patients.

THE DESPONDENT PATIENT.

JANE POTTER was admitted early one afternoon to the women's surgical ward of the London Hospital, suffering from a scalp wound caused by her husband having thrown a soda water bottle at her.

"Quarrelling?" asked the house surgeon, as he dressed the wound in the casualty room.

"Lord bless you, no," exclaimed Mrs. Potter with a forced laugh, "'E on'y threw it for fun, that's all."

When she was mounting the stairs to the ward, in company with her sister-in-law (Mrs. Pett), she remarked that the house surgeon was a rogue. "'E aint no class!" she muttered under her breath.

The next day being Sunday, Mrs. Pett set off in the afternoon to see Mrs. Potter. She found that lady, to use her own expression, "in a low state."

"Oh, dear!" moaned Mrs. Potter, "I expec' I aint long for this world. I 'eard the doctor say my temp'rature was somethink like one 'undred an' fifty, an' 'e kep' on countin' my pulse, so I reckon I'm in a bad way."

Mrs. Pett blew her nose violently and shed a few tears. "Poor soul!" she murmured, "if there's anythink you'd fancy now, I'd just slip out and get it."

"It aint any good," moaned Mrs. Potter, "I couldn't look at even an eel pie now. I feel as weak as a cat, and every now an' again my 'eart stops beatin' for quite a long time."

"Dear love! you ain't goin' ter leave us just when we'd planned that trip to Ramsgate, an' the rooms took, an' all?" sobbed Mrs. Pett.

Mrs. Potter allowed to herself that the situation was very trying, and that she was disappointed.

"I don't say," she said, after a pause, "that I might not 'ave got over this if I'd been properly took care of, but, bless your 'eart, I don't get enough to feed a rat on, an' soap an' towels is extras. Ah! well, there's more ways of killin' a cat than chokin' it with cream."

Mrs. Pett told Mr. Pett that, considering Mrs. Potter was the wife of a respectable undertaker, they "did oughter give 'er a nice tasty bit o' steak for 'er dinner, to tempt the poor dear's happetite."

Two days later, when the doctors had pronounced Mrs. Potter to be progressing favourably, Mrs. Pett found her friend "in a sinkin' state."

"What's worritin' me," groaned the patient, "is, that if I die, the children won't get the wear out of them noo red frocks."

"I should be blind if I didn't see as it would be an hexpence all round," said Mrs. Pett. "I tell you stright,' she added, "if my famerley 'as to go into mournin' jest now, we'll be slick ruined."

"I don't want Samuel to know as I 'aven't 'ad enough to heat here," said Mrs. Potter in a faint voice. "'Ed take it to 'eart so much pore man—you've on'y got to see the way 'e 'umours my fancy for pickles."

"I'll go right 'ome an' tell 'im to come 'ere 'afore it's too late," exclaimed Mrs. Pett.

"No good," replied Mrs. Potter in a resigned voice. "'E's makin' two corfins for one family—two old folks what 'ad a fancy for bein' buried together—I wouldn't spoil 'is chanst for anythink."

She laid her plump hand over her heart and breathed quickly for a few minutes. "Tell Samuel," she said with difficulty, "that if I'm to 'ave any chice in the matter, I've always fancied them lightish brown corfins—the black ones always did give me a fit o' the creeps!"

### Examination Questions for Nurses.

RULES.

THE competition is open to all. Answers must not exceed 500 words, and be written on one side of the paper only. The pseudonym, as well as the proper name and address, must be written on the *same* paper and *not on a separate sheet*. Papers may be sent in for fifteen days only from the day of the publication of the question. Failure to comply with these rules will disqualify the candidate for competition. Prizes will be awarded for the two best answers. Papers to be sent to "The Editor," with "Examination," written in the left-hand corner of the envelope.

QUESTION FOR OCTOBER.

Describe the materials used for, and the method adopted for applying, an abdominal fomentation.

### Appointments.

VICTORIA HOSPITAL, FOLKESTONE.—Miss Sarah Margaret Armitage has been appointed Matron. She was trained at the Children's Hospital, Pendlebury, and King's College, London. Subsequently she has been sister at Lewisham Infirmary and at the Hospital for Sick Children in Great Ormond Street, London.

ROYAL DEVON AND EXETER HOSPITAL, EXETER.—Miss L. Dickinson has been appointed Assistant Matron. She was trained at the Devon and Exeter Hospital, and after her probation put on the staff. Subsequently she was sister of "Summerhayes Ward" (women's surgical), which post she held until her appointment as assistant matron.

WIMBLEDON COTTAGE HOSPITAL.—Miss Lanyon has been appointed Matron. She was trained at University College Hospital, and was subsequently staff-nurse and sister. She has also been matron of Liskeard Cottage Hospital for three years.

### Minor Appointments.

SHANGHAI MUNICIPAL COUNCIL NURSING HOME.—Miss Mary J. Davies has been appointed for Private Nursing. She was trained at St. George's Hospital, the City of London Hospital, and has the L.O.S. certificate. She has since been staff-nurse in the British Hospital, Paris, and engaged in private nursing.

CITY ISOLATION HOSPITAL, NOTTINGHAM.—Miss Netta Allan has been appointed Sister. She was trained at the Town Hospital, Glasgow, and Haverstock Hill Fever Hospital, London. Subsequently she has been staff nurse at Victoria Park Hospital, London, where she acted as temporary matron and housekeeper.

SUFFOLK GENERAL HOSPITAL.—Miss Lucy Sharrock has been appointed Sister. She was trained at the Royal Albert Edward Infirmary, Wigan, and has since been charge nurse at Brook Fever Hospital, and sister of male surgical ward, Rotherham Hospital.

JAFFRAY HOSPITAL, BIRMINGHAM.—Miss Violet Paget has been appointed Staff Nurse. She was trained at the Birmingham Infirmary, and for the last year and ten months has been private nursing at the Kensington and Putney Nursing Association.

ST. JOHN'S SKIN HOSPITAL, UXBRIDGE ROAD.—Miss Dorothy Van Coppendle has been appointed Sister. She was trained at Cardiff Infirmary, and has since been taking sister's holiday duties at St. John's Hospital.

LEWISHAM INFIRMARY.—Miss Inge Bröchner has been appointed Sister. She was trained at St. Bartholomew's Hospital, and was subsequently a staff nurse.

LIVERPOOL HOSPITAL FOR CANCER AND SKIN DISEASES.—Miss Mary Rose has been appointed Sister. She was trained at Stockport Infirmary.

### Notes and Queries.

The contents of the Editor's Letter-box have now reached such unwieldy proportions that it has become necessary to establish a hard and fast rule regarding Answers to Correspondents. In future, all questions requiring replies will continue to be answered in this column without any fee. If an answer is required by letter, a fee of half-a-crown must be enclosed with the note containing the enquiry. We are always pleased to help our numerous correspondents to the fullest extent, and we can trust them to sympathise in the overwhelming amount of writing which makes the new rules a necessity.

Every communication must be accompanied by the writer's name and address, otherwise it will receive no attention.

*London Infirmaries.*

(1) Which is the best London workhouse infirmary? If there are more than one, will you give names of them and addresses.—*B. P.*

Workhouse infirmaries are numerous in London, but "The Nursing Profession: How and Where to Train" answers this question fully. Price 2s., from the Scientific Press, W.

*Maternity Training.*

(2) "Alpha" will be glad to know what are the three best hospitals or training schools where a nurse may obtain a thorough training for midwifery?

There is a long list of excellent training schools for midwifery, and it is quite impossible to say which is the best. Queen Charlotte's Hospital, Marylebone Road; the British Lying-in Hospital, Endell Street; and the General Lying-in Hospital, York Road, S.E., are the largest. At York Road preference is given to trained nurses in selecting candidates for training.

*Fees.*

(3) 1. Will you kindly tell me what are the usual charges made by a nurse for daily visits for the purpose of administering a douche, an enema, or a pack? 2. Could you recommend a book (not expensive) on the rearing of infants?—*A. S.*

1. From 2s. 6d. to 5s. a visit. 2. "The Mother's Help to the Rearing and Management of Her Children," by P. Murray Braidwood, M.D. price 2s., from the Scientific Press.

*Dispensing.*

(4) I have had a year's training in an accident hospital, and I wish to qualify myself for a dispenser. Will you kindly tell me what are the qualifications, how long it would take to learn, what it would cost, and how I should set about it?—*Cottage.*

An article on this subject appeared in "THE HOSPITAL" for July 1st last. If you wish to obtain the certificate of the Pharmaceutical Society, apply to the Secretary, 17, Bloomsbury Square, W.C., for regulations and charges.

(5) Will you tell me the best means a man can take to become a fully-qualified dispenser, the best posts to be held, both for practice and remuneration, and the usual salary given? Thanking you for past replies, and in anticipation.—*R. P.*

The best means is to apply to the Pharmaceutical Society, 17, Bloomsbury Square, W.C., for syllabus of the minor and major examinations, and then to prepare for them in one of the schools devoted to this work. The posts for qualified persons range from chemists' assistants to those attached to large institutions. The usual salary for the minor appointments is from £60 to £120.

*Abroad.*

(6) Will you kindly inform me of any institutions abroad for private nurses?—*K. P.*

"K. P." would be well advised to write for particulars to the English Consul or chaplain of the town in which she wishes to work. The Holland Institute, 1, Tavistock Chambers, W.C., has branches at various places in France. The Elgin Institute has recently opened a branch at Châlot Santa Croce, Alassio, N. Italy, whilst there are nurses' homes in Florence, Rome.

*Equality.*

(7) Would you kindly inform me if a three years' certificate from a workhouse or infirmary will be of as much use as one from a general hospital, and if the training is considered as good as any other?—*Nurse.*

The training in the best workhouse infirmaries ranks very high, and nurses trained in them are eligible for the highest posts.

*Quotation.*

(8) I have an old cutting from THE HOSPITAL of many years ago, pointing out that there were hospitals before the time of Christ—one at "Epidaurus, and dedicated to Æsculapius"—and home hospitals at Rome 250 B.C.; also that "the Saturn priests in Egypt treated people for lunacy 1500 B.C." This cutting does not give any reference to the books that have this valuable history. If it would not be troubling you too much, I should be delighted with the reference to those books, and where they are kept.—*J. K.*

It would be impossible to trace all the contributors or sources of information made use of "many years ago." You will probably obtain all the information you require in the reading-room of the British Museum.

*Monthly Work.*

(9) Will you kindly tell me if there is any co-operation where a monthly nurse (certificated), who has had some general training (not certificated) and considerable private nursing, would be accepted and employed? I should be so grateful for any information or addresses of any such co-operation.—*Monthly Nursing.*

See our advertisement columns. Such nurses are frequently wanted for the staff of private homes, though there is no association especially for them.

*Epileptic.*

(10) Could you tell me of a hospital or home where a respectable girl suffering from epileptic fits, which are sufficiently frequent to prevent her retaining a post when she has obtained it, could be taken in permanently? She is able and willing to work, but unless she can find such a place as she is seeking she will be obliged to go to the workhouse. Her father was a butcher, and the clergyman of the parish has asked me if I can help in the matter. The girl is unable to pay anything towards maintenance.—*A. R. C.*

Write to the Secretary of the National Society for the Employment of Epileptics, 12, Buckingham Street, Strand. The colony is at Chalfont St. Peters, Bucks.

*Beri-Beri.*

(11) Will you please let me know what kind of disease Beri-Beri is, and how it attacks patients?—*E. W.*

An article on Beri-Beri will shortly appear in THE HOSPITAL, from which you will be able to obtain the information you desire.

*Young Pros.*

(12) Miss Alice D. wishes to know if she is too young to be accepted as a probationer. She is not quite 21 years of age, and she is anxious to obtain some preliminary training previous to entering the London Hospital as a paying probationer.

See reply to "Aged 18."

*Aged 18.*

(13) My daughter, aged 18, desires to enter nursing profession. Will you please tell me whether any hospital (children's or otherwise) takes probationers at that age, or, if not, what is the earliest age they are received?—*M. R. H.*

You would find the "Nursing Profession: How and Where to Train," most valuable in helping you to select a training school for your daughter. The price is 2s. from the Scientific Press. It contains a list of children's hospitals, some of which will accept probationers as young as 18, though 23 is the age required by most schools.

*Pros at Cottage Hospitals.*

(14) The matron of a cottage hospital of 15 beds would be glad to have information regarding the terms on which non-paid probationers are received in hospitals of similar size. Can they be taken on a month's trial and then bound for, say, one year; and are they given uniform, and, if so, how many dresses and aprons?—*A. E.*

The matron makes what terms she likes. As a rule a month's trial is arranged before a definite period of service is fixed. Some economical matrons provide only the use of the uniform, and keep a sufficient number of frocks, aprons, and caps to serve. As worn out they are renewed, and when one probationer leaves the whole stock is passed on to her successor.

*America.*

(15) Being desirous of finding employment in United States of America as private nurse, I would be thankful if you could give me some advice as to how to obtain the same. Besides a training of three years, I have had experience in fever and maternity work, and have for the last two years been engaged in private nursing.—*Nurse Bessie.*

The United States of America is a wide field. Has Nurse Bessie any idea as to what part she would like to go? It would be best to make some definite plan before starting, as English nurses are not always welcomed there. Having fixed her destination, she could obtain reliable data from the Emigrants' Information Office, 31, Broadway, Westminster.

*Superannuation Act.*

(16) Will you kindly tell me where I can obtain a copy of the Poor Law Officers' Superannuation Act, 1896?—*L. L.*

Any law stationer will supply a copy of the Act.

*Regulations re Plague.*

(17) Will the Editor kindly inform "An Ignorant Person" as to the (1) uniform worn by the plague nurses in India; and (2) what outfit is necessary for nurses going to India?—*Nurse.*

1. All the English plague nurses in India wear Indian nursing service uniform, which is white, with scarlet collar, cuffs, and waist band, and Sister Dora caps. 2. The sum of £10 is allowed for an outfit, of which muslin blouses are a necessary feature.

*Teachers' School.*

(18) Will you kindly say if there are any training schools for teachers in sick nursing? I am a nurse, and possess certificate from one of the largest training schools in nursing, but I notice that anyone requiring an "instructress in sick nursing" lays great stress on their having experience in teaching.—*Matron.*

There is no training school for teachers of nursing in England. The sisters in the wards of the large hospitals are the teachers of the practical work, whilst the matron and medical staff arrange courses for theory. Those who lay stress on having had experience in teaching must have acquired it when in charge of a ward.

*Irish Prisons and Schools.*

In answer to "T. H. N.'s" query *re* nurses in Irish prisons, &c., if "T. H. N." will write to the Governor, Kilmainham Prison, co. Dublin, she will receive all information as to duties and salary (which I believe is very good). I do not think it would be a nice post for a lady, as her patients are nearly always very hard to manage; besides, the trained nurse has no nice company, as the women, who are called wardceresses or matrons, are generally of the servant class, and sometimes not nearly so respectable as a decent servant. With regard to "T. H. N.'s" other query, most of the large institutions, colleges, boarding schools, &c., are Roman Catholic, and are generally under the control of religious orders, so that the nuns look after the sick. In Church of Ireland schools there is always a matron (untrained of course) to superintend the domestic duties, and at the same take charge of any sick who may be in the infirmary. Ireland is too poor (and, I may add, a good deal too behind the times as regards nursing) to have the services of trained nurses to such an extent as you have in England.

### Travel Notes.

By Our Travelling Correspondent.

### XXXVII.—ROUND BRITTANY IN A FORTNIGHT.

Of late years the travelling public has become well acquainted with North Brittany, but the charming scenery, quaint costumes, and primitive customs of the Morbihan and Finisterre have been left unexplored on account of the tedious railway journey, combined with the difficulty, on arrival, of finding local trains for excursions suitable in hours to our necessities and inclinations. I am thinking now entirely of cyclists in recommending this route; for others it would require modifications. It can be done on wheels in a fortnight, and need not cost more than £8, or even less.

### The Route.

Take the Friday boat to St. Malo, arriving early next morning, have breakfast, and go on to Dinan twenty miles, sleep there, one night or two, according to time at your disposal. I shall give an article on Dinan shortly, so I will not say more now than that it is a fascinating place for artists, photographers, and for those who have time for the many delightful excursions in the surrounding country.

### Dinan to Lamballe and Pontivy.

Lamballe is distant 27 miles, and the road is good. Put up at the Hotel de France. The church of Notre Dame is worth visiting; it was originally the castle chapel, belonging to the Counts of Penthièvre, and is of the thirteenth and fourteenth centuries. Five miles outside Lamballe is the castle of La Hunaudaye, massive and frowning, with remnants of the huge round corner towers still left to attest its ancient strength. From Lamballe to Pontivy you pass Loudeac, where you can lunch, reaching Pontivy in the evening.

### Pontivy and Josselin.

The old quarter of Pontivy is supremely interesting—the streets, narrow, dark, and overhanging, lean together at the top, and from the upper windows the housewives hook their parcels from the roofs of the arriving diligences by means of the domestic tongs. Stay here for a night or two, for you must not omit a visit to Josselin, ten miles to the east. The castle, once the residence of the Constable de Clisson, is of great antiquity, and presents delightful views of the differing styles of architecture from the thirteenth to the sixteenth century. The interior I did not see, for, being told there had been extensive "improvements," my ardour for investigation was cooled.

### Guéhenno.

On your road to Vannes is Guéhenno with its wonderful Calvary. It was buried by the curé for safety in the stormy year of the Terror, and only discovered some forty years since by the reigning parish priest. Vannes contains some nice old timbered houses, but I should not recommend a lengthened stay there, and perhaps you would prefer to go straight on to Auray, which you can do by train if you prefer it.

### Auray, Carnac, and Quiberon.

You will spend some days with pleasure at Auray, for there is so much to be seen in the neighbourhood. Carnac, with its miles of Druidical stones, the singular and characteristic coastline about Lockmariaker and the Point of Quiberon, where the unfortunate Royalists under Sombreuil were literally driven into the sea in 1795, are all full of interest. You will like the Lion d'Or at Auray, where the proprietor is a most kind and attentive host.

### Quimperlé, Quimper, and the Pointe de Raz.

From Auray to Quimperlé is thirty-two miles; sleep at another Lion d'Or. This little town is curtly dismissed in the guide-books, but it is a pretty place, though not so striking as Quimper, which is the capital of Finisterre. You will probably see some remnants of costume, though it is fast disappearing before the Juggernaut of fashion, and it is becoming rare to see the men wearing the huge knickerbockers called bragoubras, and the series of embroidered waistcoats, surmounted by a sheepskin jacket. You cannot do better at Quimper than the Hotel de l'Epée, and weather permitting, lose no time in visiting the Pointe de Raz. It is the most westerly headland in Southern Brittany, and is something like our Land's End, but far wilder and more awe inspiring. To do this take the train to Douarnenez, from whence it will be a run there and back of about thirty miles. You must walk the last part of the way. Some lady friends of mine (artists) effected a lodgement in the signal station for the lighthouse, but on Sunday shared their looking-glass and toilet table with the gentleman in charge, who desired to shave and beautify himself before taking his brief holiday. The coast here is marvellous; to the north is the Baie des Trépassées, so-called from the terrible number of wrecks occurring there. In old days it was a place of evil repute, for it was a happy hunting ground for those beasts of prey, the wreckers. The town of Quimper is worth seeing; especially fine is the Cathedral, begun in 1239, the Triforium is grand, and the windows have beautiful ancient glass. From Quimper to Morlaix it is well to train; the towns you pass are of little interest and the road dull.

### Morlaix.

Over this fascinating old town I must not linger. Go to the Hotel de l'Europe and arrange for an early trip to St. Pol de Lehon and Roscoff, there and back about forty miles. The exquisite pierced spire of the Creizker at St. Pol de Lehon is the gem of the excursion. Morlaix itself is unique, and if you have artistic tastes, you will tear yourself away with difficulty. To regain St. Malo the best plan will be to wheel to Lannion, twenty-five miles, sleep a night there to visit the coast round Perros-Guirrec, then on to Guingamp, and from there train to St. Malo.

---

### TRAVEL NOTES AND QUERIES.

Rules in Regard to Correspondence for this Section.—All questioners must use a pseudonym for publication, but the communication must also bear the writer's own name and address as well, which will be regarded as confidential. All such communications to be addressed "Travel Editor, 'Nursing Mirror,' 28, Southampton Street, Strand." No charge will be made for inserting and answering questions in the inquiry column, and all will be answered in rotation as space permits. If an answer by letter is required, a stamped and addressed envelope must be enclosed, together with 2s. 6d., which fee will be devoted to the objects of the "Hospital Convalescent Fund." Any inquiries reaching the office after Monday cannot be answered in "The Mirror" of the current week.

Dieppe (Pension).—I hope this will meet your eye, but you do not give me any pseudonym. Please to read the rules at the head of this section. You ask almost an impossible thing. Dieppe is a very expensive place. There is one pension that is reasonable, but not cheap, Mrs. Buckland, Pension Anglaise, Rue Roustain, terms about three guineas a week. I think you might find the Hotel de la Paix or Hotel des Familles cheaper, if you write and make arrangements, but there is nothing really reliable and also cheap in Dieppe. If economy is essential, why not try Arques. There is an omnibus (two francs there and back); it is distant about three miles from Dieppe; two hotels, "The Château" and the Hotel d'Arques. As for Puys, it is almost a suburb of Dieppe, and partakes its prices. Try the Hotel Bellevue.

Rome (Clothes).—A[illegible]a no pseudonym. Read the rules of this section. Rome is delightful from the end of October to the middle of May. Warm clothes are needed; the sun is hot, but in the shade it is cold. Woollen underclothes should always be worn, with woollen dresses of various thickness. It is distinctly cold in the winter, but dry, clear, and very bracing. Carry an extra wrap when visiting the churches, they often strike cold as a vault after coming in from the hot sun.

Douarnenez (Planet).—No, I should not advise it as a winter residence; it is not cold, but it would be dreary. The only people calculated to enjoy the wilds of the Brittany sea coast in the winter are artists, to whom it presents endless pictures, and I do not judge from your letter that you are an artist. If you are bent upon Brittany at that time, why not try Dinard, and make excursions; or if you think that too expensive, Pont Aven, in the South, is sheltered, and the proprietors of the little hotels are accustomed to catering for artists all the year round. It is something like Barbizon in its clientèle.

Fowey (Artist).—At this season you will find the little place very full, but I judge from your letter that you are thinking of visiting it in October; it would be emptying fast then, and lodgings could be had very reasonably. Those advertised are rather expensive. Go for a night to the Ship, and look about you.

## Figures and Tables

**Figure f1:**
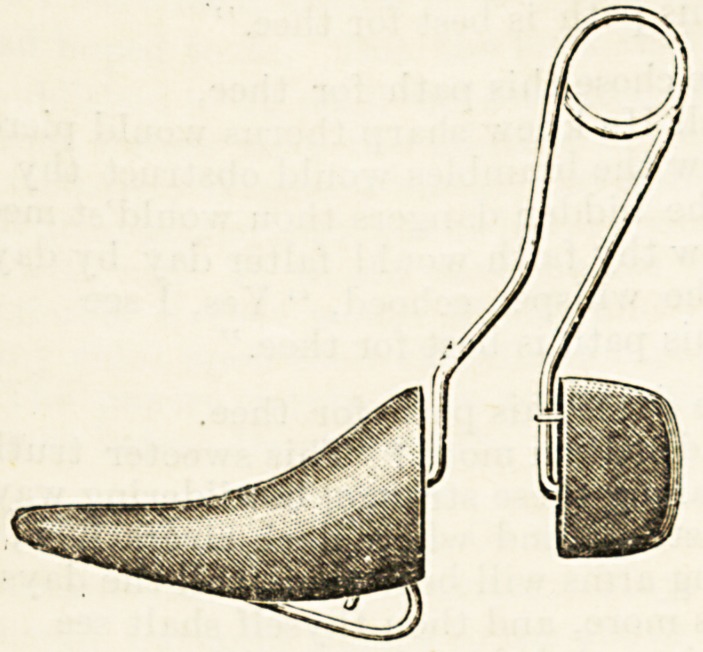


**Figure f2:**
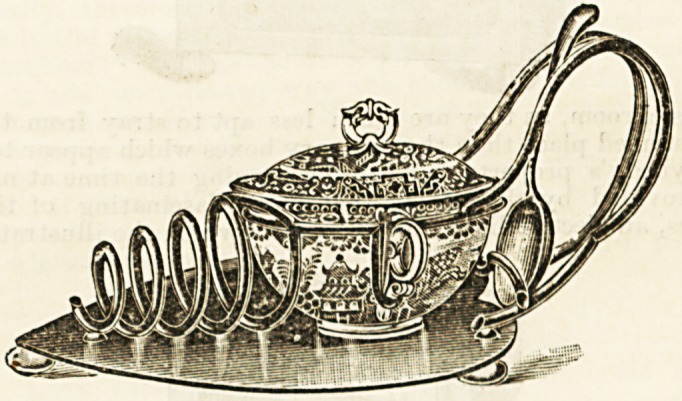


**Figure f3:**
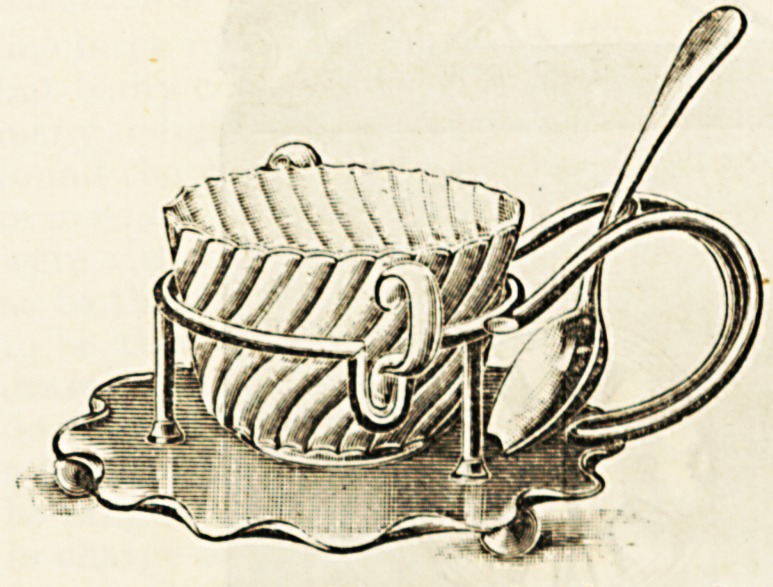


**Figure f4:**
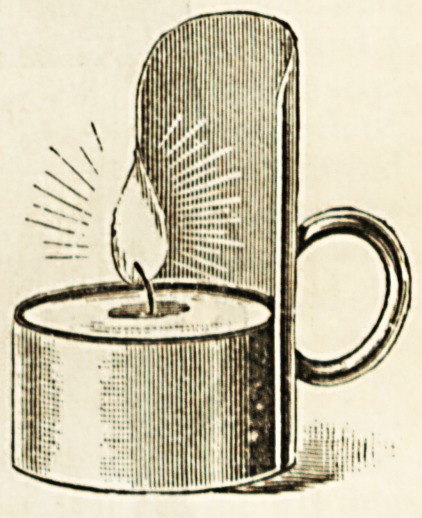


**Figure f5:**
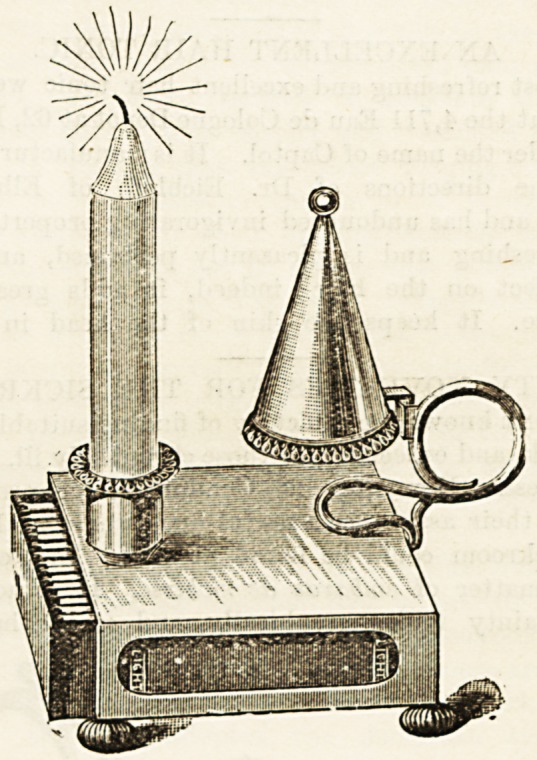


**Figure f6:**
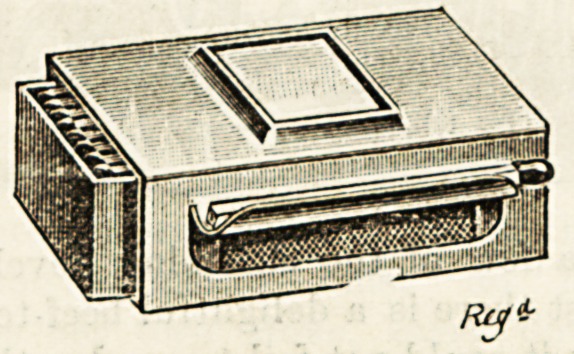


**Figure f7:**